# Intermittent compressive force promotes osteogenic differentiation in human periodontal ligament cells by regulating the transforming growth factor-β pathway

**DOI:** 10.1038/s41419-019-1992-4

**Published:** 2019-10-07

**Authors:** Jeeranan Manokawinchoke, Prasit Pavasant, Chenphop Sawangmake, Nuttapol Limjeerajarus, Chalida N. Limjeerajarus, Hiroshi Egusa, Thanaphum Osathanon

**Affiliations:** 1 Center of Excellence for Regenerative Dentistry and Department of Anatomy, Faculty of Dentistry, Chulalongkorn University, Bangkok, 10330 Thailand; 2 Division of Molecular and Regenerative Prosthodontics, Tohoku University Graduate School of Dentistry, Sendai, 980-8575 Japan; 3 Department of Pharmacology, Faculty of Veterinary Science, Chulalongkorn University, Bangkok, 10330 Thailand; 4 Research Center for Advanced Energy Technology, Faculty of Engineering, Thai-Nichi Institute of Technology, Bangkok, 10250 Thailand; 5 Department of Physiology, Faculty of Dentistry, Chulalongkorn University, Bangkok, 10330 Thailand; 6 Genomics and Precision Dentistry Research Unit, Faculty of Dentistry, Chulalongkorn University, Bangkok, 10330 Thailand

**Keywords:** Cell signalling, Osteoblasts

## Abstract

Mechanical force regulates periodontal ligament cell (PDL) behavior. However, different force types lead to distinct PDL responses. Here, we report that pretreatment with an intermittent compressive force (ICF), but not a continuous compressive force (CCF), promoted human PDL (hPDL) osteogenic differentiation as determined by osteogenic marker gene expression and mineral deposition in vitro. ICF-induced *osterix* (*OSX*) expression was inhibited by cycloheximide and monensin. Although CCF and ICF significantly increased extracellular adenosine triphosphate (ATP) levels, pretreatment with exogenous ATP did not affect hPDL osteogenic differentiation. Gene-expression profiling of hPDLs subjected to CCF or ICF revealed that extracellular matrix (ECM)-receptor interaction, focal adhesion, and transforming growth factor beta (TGF-β) signaling pathway genes were commonly upregulated, while calcium signaling pathway genes were downregulated in both CCF- and ICF-treated hPDLs. The *TGFB1* mRNA level was significantly increased, while those of *TGFB2* and *TGFB3* were decreased by ICF treatment. In contrast, CCF did not modify *TGFB1* expression. Inhibiting TGF-β receptor type I or adding a TGF-β1 neutralizing antibody attenuated the ICF-induced *OSX* expression. Exogenous TGF-β1 pretreatment promoted hPDL osteogenic marker gene expression and mineral deposition. Additionally, pretreatment with ICF in the presence of TGF-β receptor type I inhibitor attenuated the ICF-induced mineralization. In conclusion, this study reveals the effects of ICF on osteogenic differentiation in hPDLs and implicates TGF-β signaling as one of its regulatory mechanisms.

## Introduction

The periodontal ligament (PDL) is a connective tissue that links the tooth root to alveolar bone^1,2^. Fibroblasts are the main cell type residing in the PDL^3^. The PDL’s mechanoreceptors play an important role in the reflexes that prevent damage to the tooth and periodontium^4,5^. The PDL functions to resist occlusal forces, to transmit forces from the teeth to alveolar bone, to secure the teeth in the alveolar socket, and as a protective scaffold for cells, vessels, and nerves^6,7^. The PDL is exposed to various mechanical stimuli in both physiological and pathological conditions. During normal mastication, the PDL is subjected to various force types. The PDL fibers are arranged in several orientations, generating resistance to chewing forces from various directions. Compressive and tensile stresses are observed in different locations of the PDL as demonstrated by finite element analysis of simulated parafunctional and traumatically loaded teeth^8^. During orthodontic tooth movement (OTM), the PDL normally receives the force in a directional manner, resulting in a change of tooth position. Thus, mechanical force clearly participates in the regulation of PDL homeostasis.

Many publications demonstrate that mechanical force controls the biological activities and responses of cells isolated from human PDL tissue^9–11^. Cyclic stretch exposure results in differential expression of genes related to the ECM, cell adhesion, and ECM proteases in human PDL cells (hPDLs)^9^. Continuous compressive force (CCF) treatment significantly increases interleukin 6 (*IL6*), but decreases alkaline phosphatase (*ALP*), mRNA expression^10^. Hence, these results indicate the influence of different force types on hPDL behavior. Studies illustrate that CCF and intermittent compressive force (ICF) differentially regulate hPDL behavior^11,12^. CCF and ICF both significantly increased *SOST*, *TGFB1*, and *HEY1* mRNA expression in hPDLs^11^. However, the upregulation was less in the CCF-treated group compared with the ICF-treated group^11^. Furthermore, CCF treatment did not significantly alter the expression of *HES1* mRNA, while a significant upregulation of *HES1* was observed after ICF treatment^11^. Moreover, in an in vivo OTM model, CCF generated more intermediate root resorption than that of ICF application^12^. Correspondingly, histomorphometrical analysis illustrated that the percentage of osteoclast length per bone length is lower after ICF treatment compared with CCF treatment in rat molars^13^. A clinical study demonstrated that CCF application during canine retraction resulted in gradual bodily movement, while ICF led to rapid tipping of the retracted canine^14^. Both force types influenced anchorage loss in a similar manner^14^. These data imply that different mechanical forces have distinct roles in regulating hPDL responses.

Mechanical forces have been shown to regulate osteogenic differentiation in osteoblasts. However, the effect of compressive force on osteogenic differentiation potency of hPDLs remains unclear. The aim of the present study was to evaluate the effect of CCF and ICF stimuli on hPDL osteogenic differentiation. In addition, the regulatory mechanisms of ICF-pretreatment on the osteogenic differentiation of hPDLs were examined.

## Materials and methods

### Cell isolation and culture

The cell isolation protocol was approved by the Human Research Ethics Committee, Faculty of Dentistry, Chulalongkorn University (Approval number 008/2018). The inclusion criteria for the donors were: (1) age between 18‒35 years old, (2) normal healthy teeth without infection or inflammation, (3) the teeth were treatment planned for extraction, and (4) orthodontically untreated patients. Cell isolation was performed as previously described^15^. The periodontal tissues were gently scraped from the middle third of the root surface and the tissues were placed on 35-mm tissue culture dishes (cat. No. 430165, Corning, Oneonta, NY, USA) for cell explant. The explanted cells were maintained in Dulbecco’s Modified Eagle Medium (DMEM cat. No. 11960, Gibco, Grand Island, NY, USA) supplemented with 10% fetal bovine serum (cat. No. 10270, Gibco), 1% l-glutamine (GlutaMAX^TM^−1, cat. No. 35050, Gibco), and 1% Antibiotic-Antimycotic (penicillin, streptomycin, amphotericin B, cat. No. 15240, Gibco). The cells were incubated at 37 °C in a humidified 5% carbon dioxide atmosphere. The culture medium was changed every 48 h. After reaching confluence, the cells were subcultured at a 1:3 ratio using a trypsin/EDTA solution (cat. No. 25200, Gibco).

### Compressive force treatment

The compressive force treatment was performed using a computer-controlled apparatus^11,16^. Cells were seeded in 6-well tissue culture plates (cat. No. 430166, Corning) at a density of 37,500 cells/cm^2^ for 24 h and the cells were then serum starved for 8 h prior to compressive force treatment. The compressive force application parameters were as previously described^11,16^. Briefly, an ICF was applied on the cells with a loading frequency of 0.23 Hz at a 1.5 g/cm^2^ force. The CCF was applied using continuous loading with a 1.5 g/cm^2^ force. The mechanical stimulation was performed in serum-free culture medium. In some conditions, the cells were pretreated with TGF-β receptor type 1 inhibitor (SB431542 4 μM, cat. No. S4317, Sigma-Aldrich, St. Louis, MO, USA), TGF-β1 neutralizing antibody (5 μg/ml, cat. No. MAB240, R&D Systems, Minneapolis, MN, USA), suramin (15 μM, cat. No. 574625, Calbiochem^®^, La Jolla, CA, USA), JNK inhibitor (40 nM, cat. No. 420119, Calbiochem^®^), p38 MAP kinase inhibitor (35 nM, cat. No. 506126, Calbiochem^®^), cycloheximide (1 μg/ml, cat. No. C-0934, Sigma-Aldrich), Rho-kinase inhibitor (12.7 nM, cat. No. 555550, Calbiochem^®^), or monensin (1 μM, cat. No. M5273, Sigma-Aldrich) for 30 min prior to mechanical force treatment.

### Osteogenic differentiation

Cells were seeded on 6-well tissue culture plates at a density of 37,500 cells/cm^2^. The cells were subjected to CCF or ICF stimulation in serum-free medium for 24 h. Subsequently, the culture medium was changed to osteogenic medium, which was normal growth medium supplemented with β-glycerophosphate (5 mM, cat. No. G9422, Sigma-Aldrich), l-ascorbic acid (50 μg/mL, cat. No. A-4034, Sigma-Aldrich), and dexamethasone (250 nM, cat. No. D8893, Sigma-Aldrich). The medium was changed every 48 h.

In other experiments, cells were seeded on 48-well tissue culture plates (cat. No. 3548, Costar^®^, Corning) at a density of 37,500 cells/cm^2^ and allowed to attach for 24 h. The cells were then starved in serum-free medium for 8 h and subsequently exposed to adenosine 5′-triphosphate disodium salt hydrate (ATP, cat. No. A6419, Sigma-Aldrich) or recombinant human TGF-β1 (cat. No. 616455, Calbiochem^®^) for 24 h in serum-free culture medium. The cells were then maintained in osteogenic medium.

### Alizarin Red S staining

Mineral deposition was determined using alizarin red s staining. Briefly, the samples were fixed with ice-cold methanol (cat. No. 100230, Honeywell, Ulsan, Korea) for 10 min. The mineral deposits were stained with 1% alizarin red s (cat. No. A5533, Sigma-Aldrich) solution for 3 min at room temperature. The samples were gently rinsed with deionized water between procedures. The stained mineral deposits were solubilized in 10% cetylpyridinium chloride solution and the optical density was measured at 570 nm. The relative absorbance was calculated by normalizing the results to the corresponding controls.

### ATP assay

The amount of extracellular ATP after mechanical stimuli was determined using an ENLITEN^®^ ATP assay system bioluminescence detection kit (cat. No. FF2000, Promega, Madison, WI, USA). Briefly, aliquots of culture media (50 μL) were added to the microplate. Subsequently 50 μL of Enliten Luciferase/Luciferin solution was added and the signal was immediately measured using a microplate reader (Synergy H1, Biotek multi-mode reader, Winooski, VT). The amount of extracellular ATP was quantified using a standard curve and the value was normalized to the control of each donor.

### Immunofluorescence staining

The cells were fixed with 4% buffered formalin (cat. No. F-1268, Sigma-Aldrich) for 10 min and permeabilized with 0.1% Triton-X100 (cat. No. 22686, USB corporation, Cleveland, OH, USA) for 5 min. Nonspecific binding was blocked by incubating the cells with 2% horse serum (cat. No. SH30074, Hyclone, South Logan, UT, USA) for 30 min. The specimens were then incubated with primary antibody at 4 °C overnight. The primary antibodies used in the present study were rabbit anti-osterix (OSX) antibody (1000× dilution, cat. No. ab22552, Abcam, Cambridge, UK) and mouse anti-TGF-β1 antibody (10x dilution, cat. No. MAB240, R&D Systems). A biotinylated goat-anti rabbit antibody (1000× dilution, cat. No. sc-2040, Santa Cruz Biotechnology, Dallas, TX, USA) or a biotinylated goat-anti mouse antibody (1000× dilution, cat. No. B2763, Lifetechnologies^TM^, Eugene, OR, USA) were employed as secondary antibodies. Streptavidin-FITC (cat. No. S3762, Sigma-Aldrich) was used for fluorescence labeling. The specimens were incubated with secondary antibodies and Strep-FITC for 40 min each. The specimens were gently washed with PBS between each step. DAPI (cat. No. 5748, TOCRIS bioscience, Bristol, UK) was used to counterstain the nuclei. The specimens were then observed under an Apotome.2 (Carl Zeiss, Jena, Germany) fluorescence microscope.

### RNA sequencing

After compressive force treatment for 24 h, total RNA was isolated using an RNeasy Plus Mini Kit (cat. No. 74134, Qiagen, Germantown, MD, USA). RNA sequencing was performed at the Omics Science and Bioinformatics Center, Faculty of Science, Chulalongkorn University. Briefly, total RNA quality was examined using an Agilent 2100 BioAnalyzer (Agilent Technologies, CA, USA). Sequencing libraries were constructed using a TrueSeq mRNA stranded library prep kit (Illumina, CA, USA). Subsequently, the library quality was determined using an Agilent 2100 BioAnalyzer and Qubit 3.0 fluorometer (Thermo Fisher Scientic, MA, USA). RNA sequencing was performed using a NextSeq 500 (Illumina). The reads quality was checked, trimmed, and filtered by FastQC and Trimmomatic^17,18^. Read mapping was performed using HISAT2 against *Homo sapiens* UCSC hg38^19^. Transcript quantification was performed using HTseq count^20^. For data filtering, the genes with the lowest 15% variance based on inter-quartile range and the genes with less than 4 counts in total were removed. Differential gene expression was determined using EdgeR^21,22^. Differentially expressed genes that exhibited an at least twofold up- or downregulation were included. A significant difference was defined as false-discovery rate < 0.05. The raw data was deposited in the NCBI Sequence Read Archive (SRP136155) and NCBI Gene Expression Omnibus (GSE112122). Gene ontology and enriched pathways were analyzed using WebGestalt and Reactome^23,24^.

### Polymerase chain reaction

Total cellular RNA was isolated using RiboEx^TM^ solution (cat. No. 301-001, GeneAll^®^, Seoul, South Korea). The isolated RNA integrity and amount was examined using a Nanodrop2000 (Thermo Scientific, USA). The absorbance ratios at 260/280 and 260/230 nm were evaluated. Subsequently, RNA (1 μg) was converted to complimentary DNA using an ImProm-II^TM^ Reverse Transcription System (cat. No. A3800, Promega). One microliter of complimentary DNA was employed for real-time polymerase chain reaction using a FastStart Essential DNA Green Master kit (cat. No. 06402712001, Roche Diagnostics, Mannheim, Germany). The reaction was performed on a LightCycler^®^ 96 real-time polymerase chain reaction (PCR) system (Roche Diagnostics). Relative gene expression was calculated using the 2^−ΔΔCt^ method^25^. According to our preliminary data, we evaluated the expression stability of three reference genes: *GAPDH, 18S RNA*, and *ACTB. GAPDH* was chosen as reference gene. The expression value was normalized to the *GAPDH* expression value and the control. The oligonucleotide primers are shown in Supplementary Table 1.

### Enzyme linked immunosorbent assay (ELISA)

Cell lysate was extracted using RIPA buffer. Conditioned medium was collected for measuring TGF-β1 levels using a human TGF-β1 immunoassay kit (cat. No. DB100B, R&D Systems). ELISA was performed per the manufacturer’s instructions. The optical density was measured at 450 nm. The amount of TGF-β1 was calculated using a standard curve and subsequently normalized to total protein and the control condition.

### Western blot

Protein extraction was performed using RIPA buffer containing a protease inhibitor cocktail (cat. No. P8340, Sigma–Aldrich). The protein samples were electrophoresed on a 12% sodium dodecyl sulfate–polyacrylamide gel and then transferred onto nitrocellulose membranes. The membranes were incubated with primary antibody at 4 °C overnight. The following primary antibodies were used: (i) a rabbit polyclonal antibody to SP7/osterix (1000× dilution, cat. No. ab22552; Abcam), and (ii) a mouse IgG against GAPDH (2000x dilution, MAB374, MILLIPORE, Temecula, CA, USA). A biotinylated secondary antibody (1000× dilution) was subsequently incubated with the membrane. The following secondary antibodies were used: (i) biotin conjugated goat anti-rabbit antibody (cat. No. sc-2040, Santa Cruz); or (ii) biotin conjugated goat-anti mouse antibody (cat. No. B2763, Lifetechnologies^TM^). Subsequently, membranes were incubated with peroxidase-labeled streptavidin. The signal was visualized by chemiluminescence (SuperSignal^®^ West Pico Chemiluminescent Substrate; cat. No. 34079; Thermo SCIENTIFIC, Rockford, IL, USA). The band density was determined using ImageJ software. Band density of target proteins was normalized to band density of GAPDH and subsequently normalized to the control.

### Statistical analyses

Data presentation and statistical analyses were performed using Prism 8 (GraphPad Software, CA, USA). The data are shown as mean ± standard error of mean (SEM). SEM was calculated based on the individual number of different donors employed in each experiment. Cells from at least four different donors without pooling were employed in each experiment. For RNA sequencing and western blot analysis, cells from three different donors were employed. Each dot in the graph represents the value from the independent experiments. For two independent group comparisons, the Mann Whitney *U* test was employed. Kruskal Wallis followed by pairwise comparison was used to determine significant differences for three or more group comparison. Correction of multiple comparisons using statistical hypothesis testing was performed by Dunn’s test with Prism 8 software. Significance was considered at *p* < 0.05.

## Results

### ICF pretreatment-induced hPDL osteogenic differentiation

**Fig. 1:**
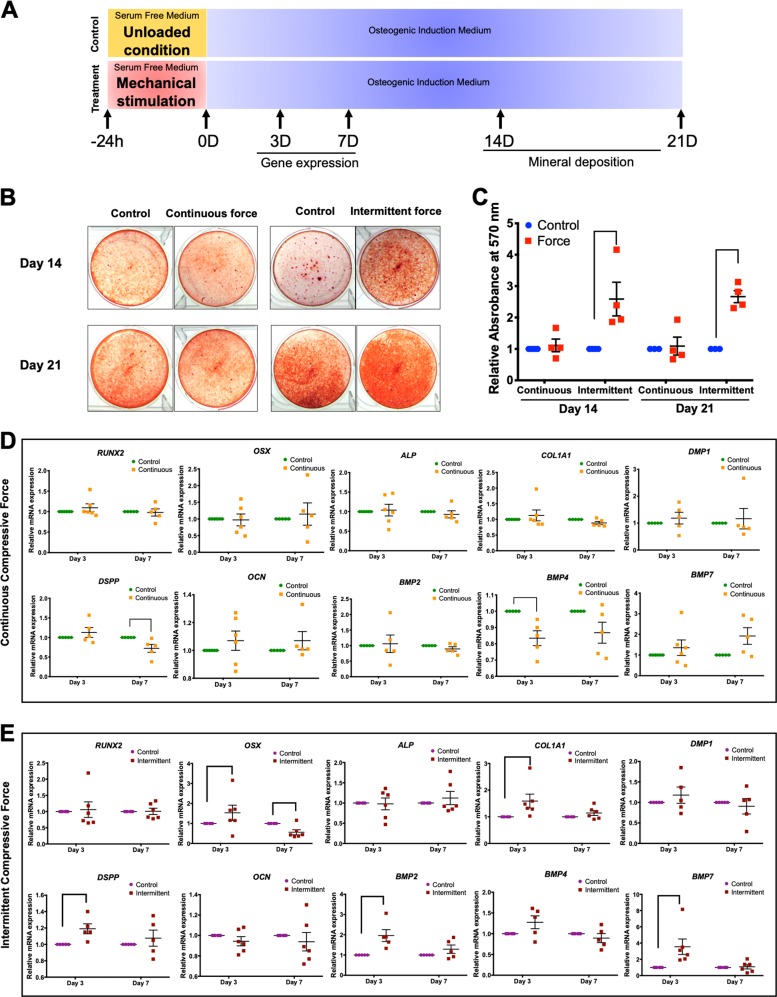
ICF stimulated osteogenic differentiation in hPDLs. Cells were exposed to CCF or ICF in serum-free media for 24 h and subsequently maintained in osteogenic medium (**a**). In the control condition, cells were cultured in serum-free culture medium for 24 h without mechanical loading and further maintained in osteogenic medium (**a**). Mineral deposition shown by alizarin red s staining at 14 and 21 days after osteogenic induction (**b**). The relative absorbance of the solubilized alizarin red dye was demonstrated (**c**). After pretreating cells with the CCF (**d**) or ICF (**e**), osteogenic marker gene expression was evaluated compared with the unloaded control using real-time polymerase chain reaction at 3 and 7 days after osteogenic induction. Bars indicate a significant difference between conditions

Cells were pretreated with CCF or ICF for 24 h in serum-free culture medium and subsequently maintained in osteogenic medium for 21 days (Fig. 1a). In the control condition, cells were cultured in serum-free culture medium for 24 h without mechanical loading and further maintained in osteogenic medium (Fig. 1a). The cells pretreated with ICF demonstrated markedly enhanced mineral deposition compared with the control at both day 14 and day 21 after osteogenic induction (Fig. 1b, c). However, CCF pretreatment did not significantly influence mineral deposition at either time point (Fig. 1b, c).

Pretreatment with CCF did not significantly influence the mRNA expression of *RUNX2*, *OSX*, *ALP*, *COL1A1*, *DMP1*, *OCN*, *BMP2*, or *BMP7* (Fig. 1d). Reduced *BMP4* and *DSPP* mRNA levels were observed at day 3 and 7 in osteogenic medium, respectively. hPDLs pretreated with ICF significantly upregulated *OSX*, *COL1A1*, *DSPP*, *BMP2*, and *BMP7* mRNA expression at day 3 of osteogenic induction (Fig. 1e). There was no significant difference in *RUNX2*, *ALP*, *DMP1*, *OCN*, or *BMP4* mRNA levels between ICF-pretreated cells and the control cells. These results imply the promotion of osteogenic marker gene expression in osteogenic inductive condition of ICF-pretreated hPDLs.

**Fig. 2:**
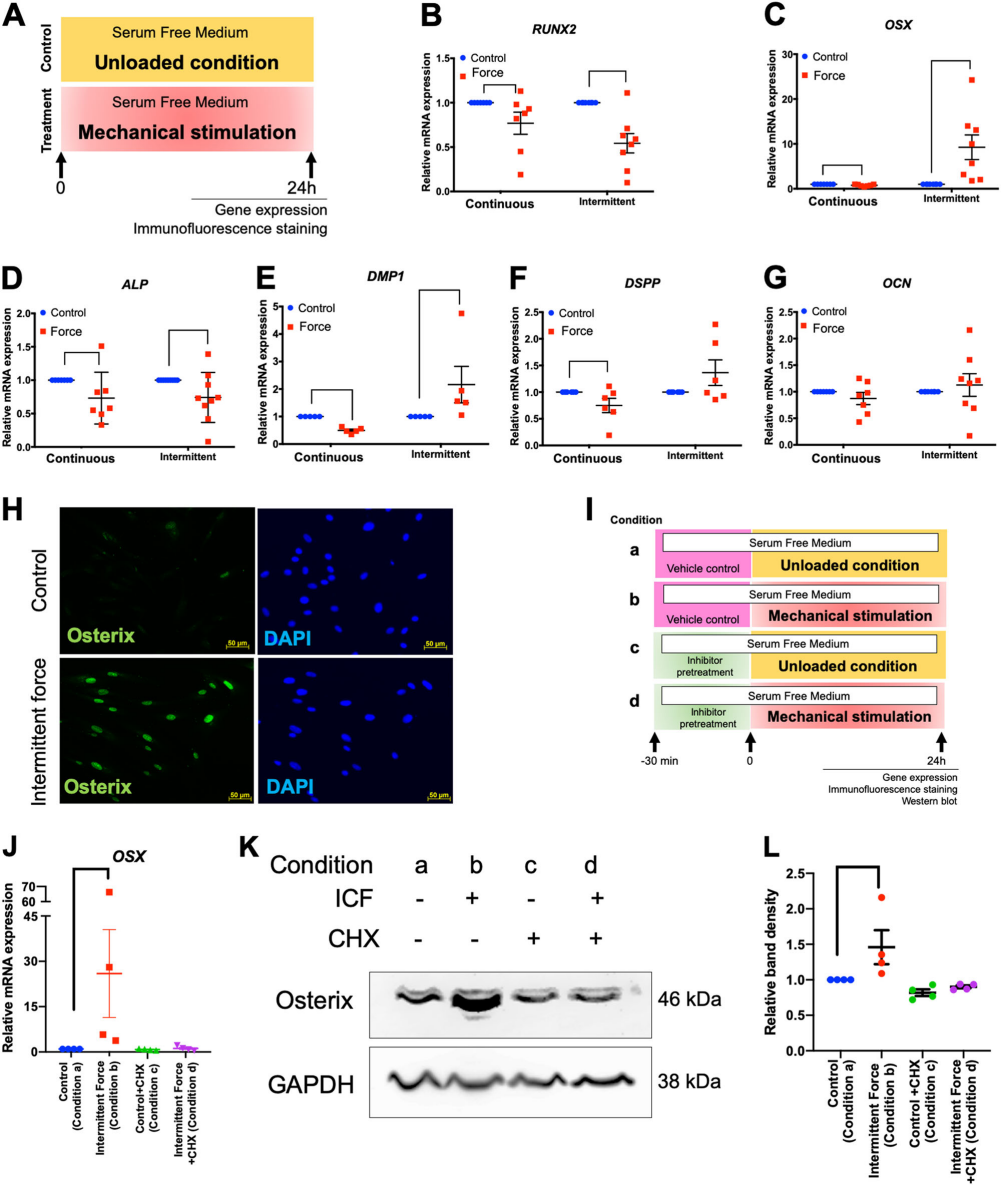

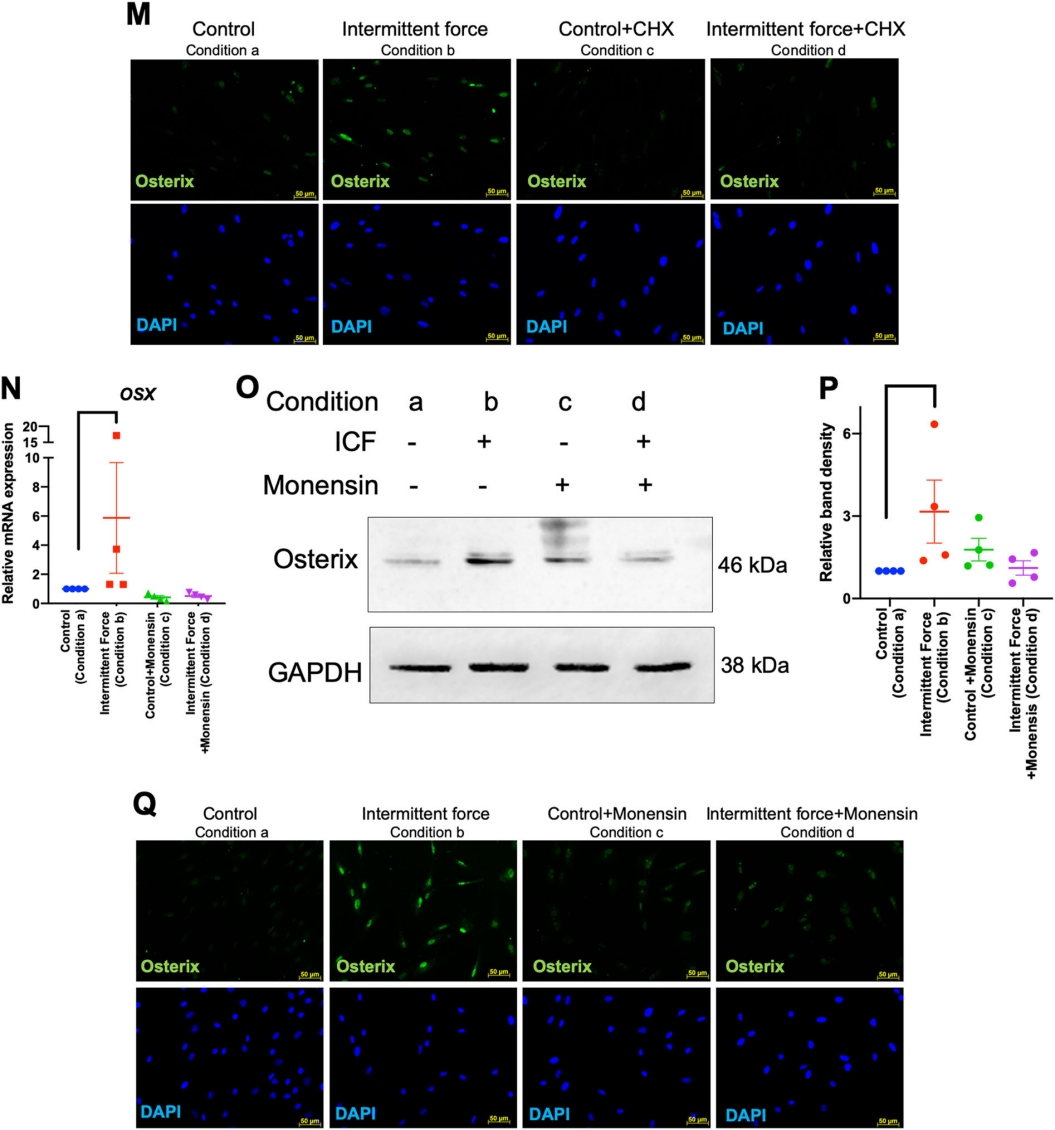
ICF induced OSX expression in hPDLs. Cells were treated with CCF or ICF in serum-free media for 24 h and cells cultured in the same condition without mechanical force application were used as the control (**a**). The mRNA expression of osteogenic marker genes was examined using real-time polymerase chain reaction (**b–g**). OSX protein expression was evaluated using immunofluorescence staining (**h**). In the inhibition experiments, cells were pretreated with inhibitor 30 min prior to ICF stimulation. Experimental conditions were illustrated (**i**). The *OSX* mRNA (**j**, **n**) were determined using real-time polymerase chain reaction. Osterix protein expression was evaluated using western blot (**k**, **o**) and the normalized band density was demonstrated (**l**, **p**). In addition, osterix protein expression was also determined by immunofluorescence staining (**m**, **q**). Bars indicate a significant difference between conditions. Scale bars indicate 50 μm

Cells were stimulated with CCF or ICF in serum-free medium for 24 h (Fig. 2a). Cells cultured in the same condition without mechanical force application were used as the control (Fig. 2a). The results demonstrated that CCF treatment significantly reduced *RUNX2*, *OSX*, *ALP*, *DMP1*, and *DSPP* mRNA expression in hPDLs (Fig. 2b–f). In contrast, ICF treated cells exhibited significantly increased *OSX* and *DMP1*, but reduced *RUNX2* and *ALP*, mRNA expression (Fig. 2b–f). *OCN* mRNA levels were not altered by either CCF or ICF treatment at 24 h (Fig. 2g).

ICF-induced OSX protein expression was confirmed using immunofluorescence staining (Fig. 2h). In the chemical inhibitor experiment, the experimental condition was demonstrated in Fig. 2i. Pretreatment with cycloheximide or monensin 30 min prior to ICF stimulation abolished the force-induced *OSX* mRNA and protein expression in hPDLs (Fig. 2j–q).

### ATP priming did not influence hPDL osteogenic differentiation

**Fig. 3:**
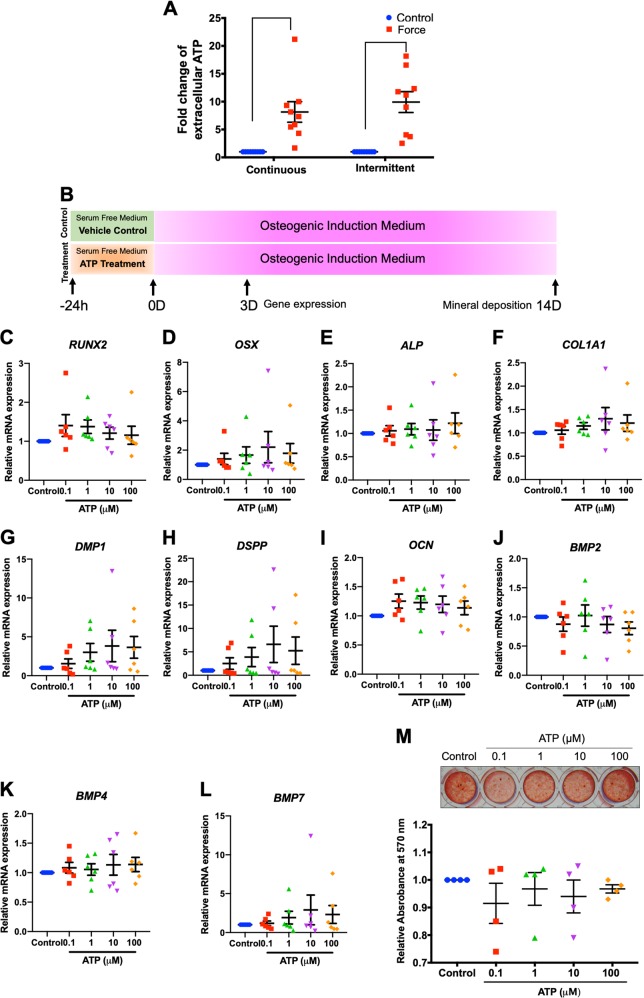
ATP priming did not influence osteogenic differentiation in hPDLs. Cells were treated with CCF or ICF in serum-free media for 24 h. Extracellular ATP was evaluated using an ATP assay in culture medium (**a**). Schematic diagram of the experimental plan of the ATP priming was illustrated (**b**). Cells were exposed to ATP for 24 h in serum-free medium. Thereafter, the culture medium was changed to osteogenic medium. Osteogenic marker gene expression was determined using real-time polymerase chain reaction at day 3 after osteogenic induction (**c**–**l**). Mineral deposition was examined using alizarin red s staining at day 14 (**m**). The normalized absorbance of the solubilized dye. Bars indicate a significant difference between conditions

A previous report showed that compressive force stimulated adenosine triphosphate (ATP) release in hPDLs^26^. In addition, it has been demonstrated that low levels of ATP induced osteogenic differentiation in human osteoblast-like cells^27^. Hence, we hypothesized that ICF enhanced osteogenic differentiation in hPDLs via ATP release. To test this hypothesis, cells were treated with CCF or ICF for 24 h in serum-free media and the concentration of extracellular ATP was determined. The extracellular ATP concentration was significantly increased in both CCF- and ICF-treated conditions compared with the unloaded control (Fig. 3a). The fold-change of extracellular ATP of the cells treated with CCF or ICF was comparable. To evaluate if the short-term release of extracellular ATP was involved with osteogenic differentiation, cells were pretreated with ATP (0.1‒100 μM) for 24 h in serum-free medium and subsequently maintained in osteogenic medium (Fig. 3b). In control condition, cells were maintained in serum-free medium for 24 h without ATP supplementation and further maintained in osteogenic medium (Fig. 3b). The short-term exposure of 0.1‒100 μM ATP did not significantly influence osteogenic marker gene expression at day 3 of osteogenic induction (Fig. 3c–l). Correspondingly, there was no significant difference in mineral deposition between the ATP treated cells and the control as evaluated by alizarin red s staining at 14 days after osteogenic induction (Fig. 3m).

### Differential gene-expression profiling of CCF- and ICF-treated hPDLs

**Fig. 4:**
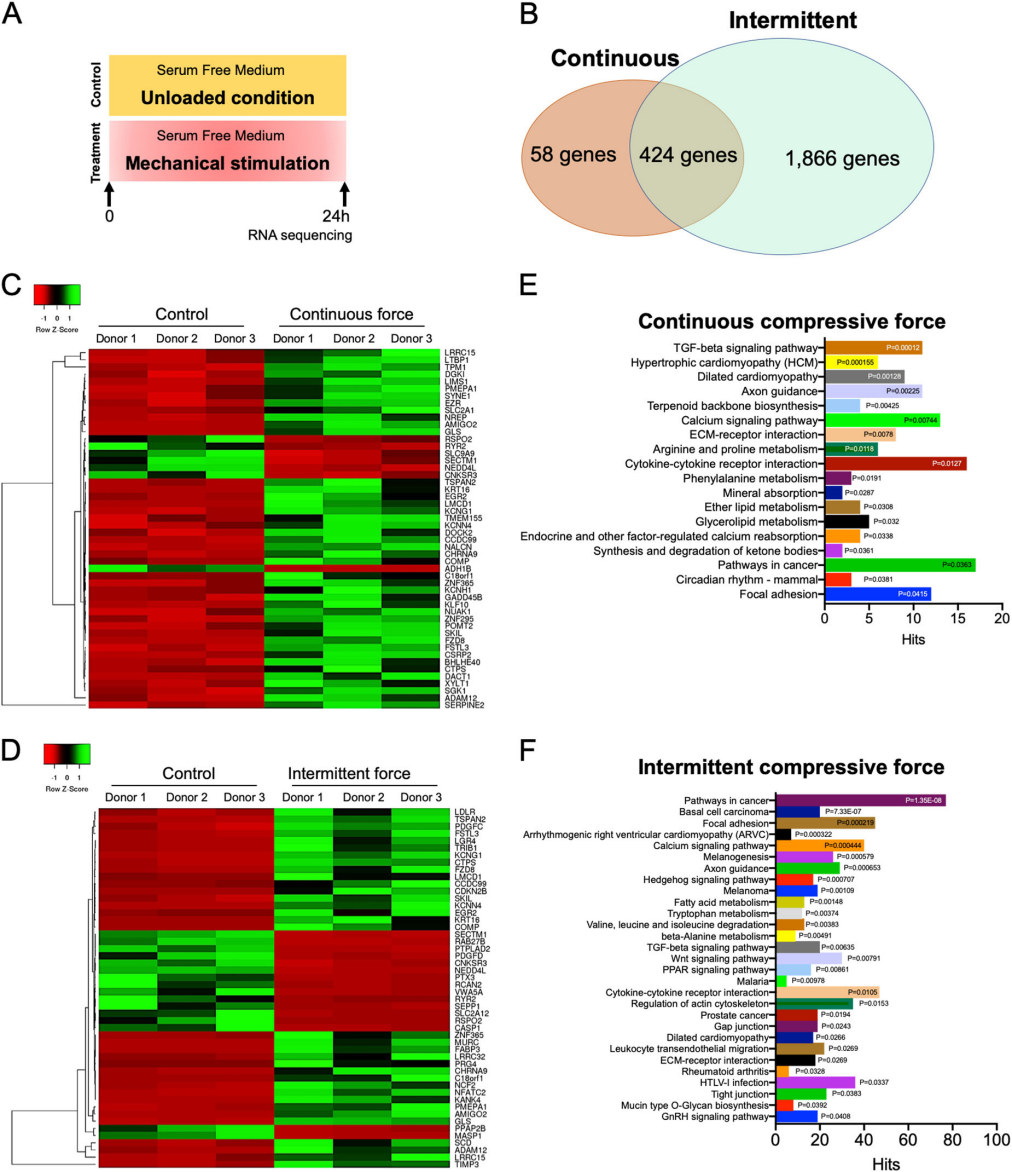
Gene expression profiling of mechanical force treated hPDLs. Cells were treated with CCF or ICF in serum-free medium for 24 h. Cells maintained in serum-free medium without mechanical loading were employed as the control (**a**). The gene expression profile compared with the unloaded control was determined using RNA sequencing and bioinformatic analyses. Common differentially expressed genes in the CCF- or ICF-treated hPDLs are shown (**b**). Heat maps demonstrating the top 50 differentially expressed genes in the CCF- (**c**) or ICF- (**d**) treated cells compared with the unloaded control. Pathway enrichment of all differentially expressed genes using KEGG database revealed the differentially regulated pathways after CCF (**e**) and ICF (**f**) treatment

To further identify pathway(s) regulating ICF-induced osteogenic differentiation, hPDLs were serum starved and subsequently exposed to CCF or ICF for 24 h in serum-free medium. Cells maintained in serum-free medium without mechanical loading were employed as the control (Fig. 4a). Total RNA was collected and subjected to RNA sequencing analysis for differential mRNA expression profiling. The control cultures were maintained in the same condition without mechanical stimulation. The results demonstrated that CCF- and ICF-treated hPDLs exhibited 482 and 2290 differentially regulated genes, respectively. Among these differentially expressed genes, 424 genes were found in both hPDLs loaded with CCF or ICF (Fig. 4b). Heatmaps of the top 50 differentially expressed genes in CCF- or ICF-treated hPDLs are illustrated in Fig. 4c, d. The top 20 significantly upregulated and downregulated genes in CCF- or ICF-treated hPDLs are listed in Supplementary Tables 2 and 3.

The enriched KEGG pathways in CCF- or ICF-treated hPDLs are presented in Fig. 4e, f as well as Supplementary Tables 4 and 5. In Fig. 4e, f, all differentially expressed genes were included for enrichment analyses. To investigate further in detail, the differentially expressed genes were categorized into upregulated and downregulated genes. Subsequently, KEGG pathway enrichment analysis was separately performed for upregulated and downregulated genes (Supplementary Tables 4 and 5). Among the upregulated enriched pathways, the upregulated genes in the CCF-treated cells were found in the ECM-receptor interaction and transforming growth factor β (TGF-β) signaling pathway genes (Supplementary Table 4). Moreover, CCF treatment downregulated genes in the calcium signaling pathway and cytokine–cytokine receptor pathway. In ICF-treated hPDLs, focal adhesion, regulation of actin cytoskeleton, TGF-β signaling pathway, and cytokine–cytokine receptor pathway genes were found among the upregulated enriched pathways (Supplementary Table 5). The downregulated genes in ICF-treated hPDLs were involved in the calcium signaling pathway. Focal adhesion and TGF-β signaling pathway genes were upregulated, while the calcium signaling pathway was downregulated in both CCF- and ICF-treated hPDLs (Supplementary Tables 4 and 5). Interestingly, the Wnt signaling pathway genes were upregulated by ICF-treatment, but not by CCF stimulation (Supplementary Tables 4 and 5).

### CCF and ICF differentially regulated ECM-receptor interaction, focal adhesion, and TGF-β signaling pathway genes in hPDLs

**Fig. 5:**
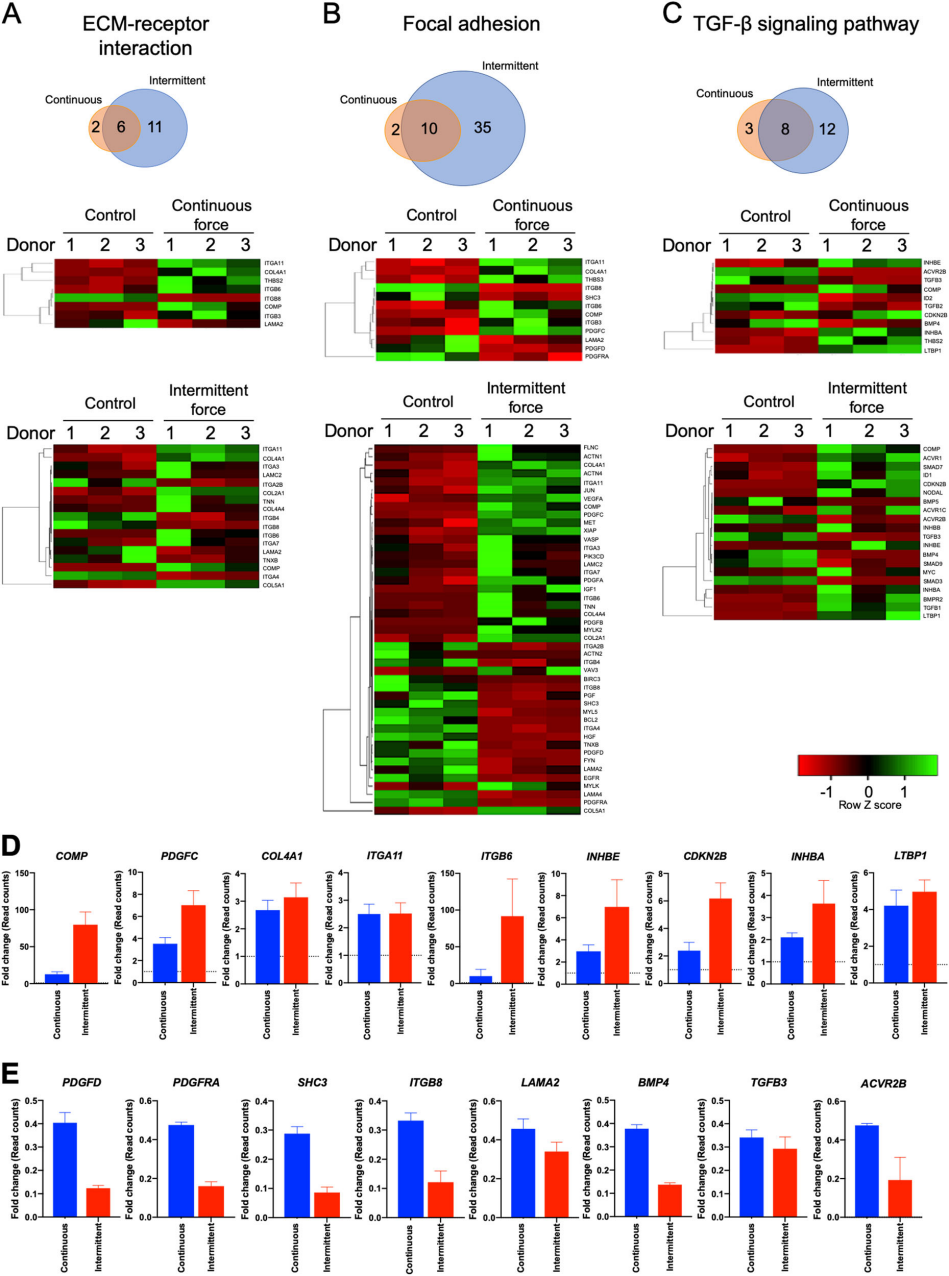
Mechanical force regulated ECM-receptor interaction, focal adhesion, and TGF-β signaling pathways in hPDLs. Bioinformatic analysis demonstrated the common differentially expressed genes and heat maps in ECM-receptor interaction (**a**), focal adhesion (**b**), and TGF-β signaling (**c**) pathways. The significantly upregulated (**d**) and downregulated (**e**) genes were selected. The fold-change of the raw read counts from the RNA sequencing data were presented. Dotted lines indicate the normalized expression value of the unloaded control

CCF differentially regulated the expression of 8, 12, and 11 genes, while ICF regulated 17, 45, and 20 genes in ECM-receptor interaction, focal adhesion, and TGF-β signaling pathways, respectively. Six, ten, and eight commonly regulated genes in both CCF- and ICF-treated cells were noted in ECM-receptor interaction, focal adhesion, and TGF-β signaling pathways, respectively (Fig. 5a–c).

In the TGF-β signaling pathway, CCF induced the mRNA expression of *CDKN2B*, *INHBE*, *THBS2*, *LTBP1*, *COMP*, and *INHBA*, but suppressed *ID2*, *BMP4*, *ACVR2B*, *TGFB2*, and *TGFB3* expression (Fig. 5c). ICF regulated the mRNA expression of 20 genes involved in TGF-β signaling. The upregulated genes are *TGFB1*, *CDKN2B*, *LTBP1*, *ID1*, *ACVR1*, *BMPR2*, *INHBB*, *ACVR1C*, *MYC*, *SMAD7*, *INHBE*, *COMP*, *INHBA*, and *NODAL*, while the downregulated genes are *BMP5*, *ACVR2B*, *TGFB3*, *BMP4*, *SMAD9*, and *SMAD3*.

The common genes regulated by both CCF and ICF in ECM-receptor interaction, focal adhesion and TGF-β signaling pathways were illustrated in Fig. 5d, e. The fold-change of the raw read count from RNA sequencing was plotted to compare the expression of the common genes between both force types. ICF induced a dramatic fold-change in *COMP*, *PDGFC*, *ITGB6*, *INHBE*, *CDKN2B*, *INHBA*, *PDGFD*, *PDGFRA*, *SHC3*, *ITGB8*, *LAMA2*, *BMP4*, and *ACVR2B* expression compared with CCF (Fig. 5d, e). The expression of *COL4A1*, *ITGA11*, *LTBP1*, and *TGFB3* mRNA was comparable between cells treated with CCF or ICF.

### ICF-regulated TGF-β1 expression in hPDLs

**Fig. 6:**
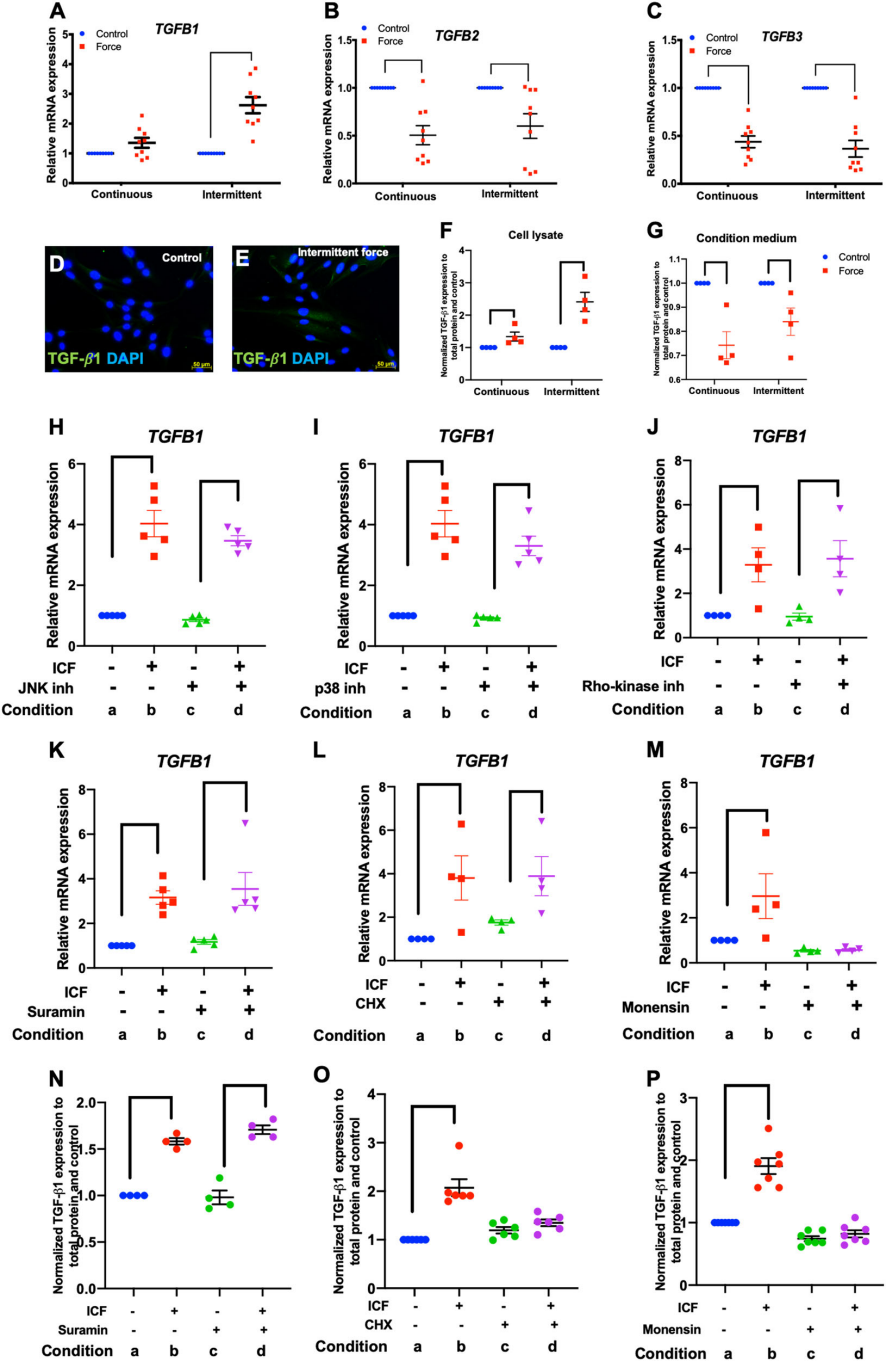
ICF induced TGF-β1 expression. Cells were treated with CCF or ICF in serum-free media for 24 h. Cells cultured in the same condition without mechanical force application were used as the control. Experimental conditions were illustrated in Fig. 2a. The mRNA expression of *TGFB1* (**a**)*, TGFB2* (**b**), and *TGFB3* (**c**) was evaluated using real-time polymerase chain reaction. TGF-β1 protein expression was examined using immunofluorescence staining (**d**, **e**) and enzyme linked immunosorbent assay (**f**, **g**). To investigate the regulatory pathways, cells were pretreated chemical inhibitor 30 min prior to ICF treatment. Experimental conditions were illustrated in Fig. 2i. Chemical inhibitors were JNK inhibitor (**h**), p38 inhibitor (**i**), Rho-kinase inhibitor (**j**), suramin (**k**, **n**), cycloheximide (**l**, **o**), or monensin (**m**, **p**). *TGFB1* mRNA and protein levels were examined using real-time polymerase chain reaction and enzyme linked immunosorbent, respectively. Bars indicate a significant difference between conditions. ICF intermittent compressive force treatment, CHX cycloheximide. Scale bars indicate 50 μm

Real-time PCR was performed on selected genes in the TGF-β signaling pathway to validate the RNA sequencing data. Cells were treated with CCF or ICF in serum-free medium for 24 h. Cells cultured in the same condition without mechanical force application were used as the control. Experimental design was illustrated as in Fig. 2a. The results demonstrated that *TGFB1* was differentially regulated by CCF and ICF (Fig. 6a). ICF significantly increased *TGFB1* expression, while CCF did not significantly alter *TGFB1* mRNA levels compared with the unloaded control (Fig. 6a). Both CCF and ICF significantly inhibited *TGFB2* and *TGFB3* expression (Fig. 6b, c), confirming the results from the RNA sequencing experiment. Immunofluorescence staining revealed that ICF markedly induced TGF-β1 protein expression compared with the unloaded control (Fig. 6d, e). The TGF-β1 protein levels in cell lysates and condition medium were also examined (Fig. 6f, g). ICF markedly induced TGF-β1 expression compared with CCF in cell lysates. However, the TGF-β1 expression was decreased in the conditioned medium from the compressive force treatment condition.

We further investigated the regulatory mechanism by which ICF promoted *TGFB1* mRNA expression in hPDLs. Cells were pretreated with chemical inhibitors for 30 min before being exposed to ICF. The results demonstrated that pretreatment with a JNK inhibitor, p38 inhibitor, Rho-kinase inhibitor, cycloheximide, and suramin did not affect the ICF-induced *TGFB1* mRNA expression (Fig. 6h–l). Interestingly, monensin abolished the effect of ICF on *TGFB1* expression (Fig. 6m). Similar to mRNA expression, suramin pretreatment did not alter ICF-induced TGF-β1 protein levels (Fig. 6n). However, cycloheximide and monensin pretreatment inhibited the effect of ICF on TGF-β1 expression (Fig. 6o, p).

### TGF-β1 participated in the ICF-induced hPDL osteogenic induction

**Fig. 7:**
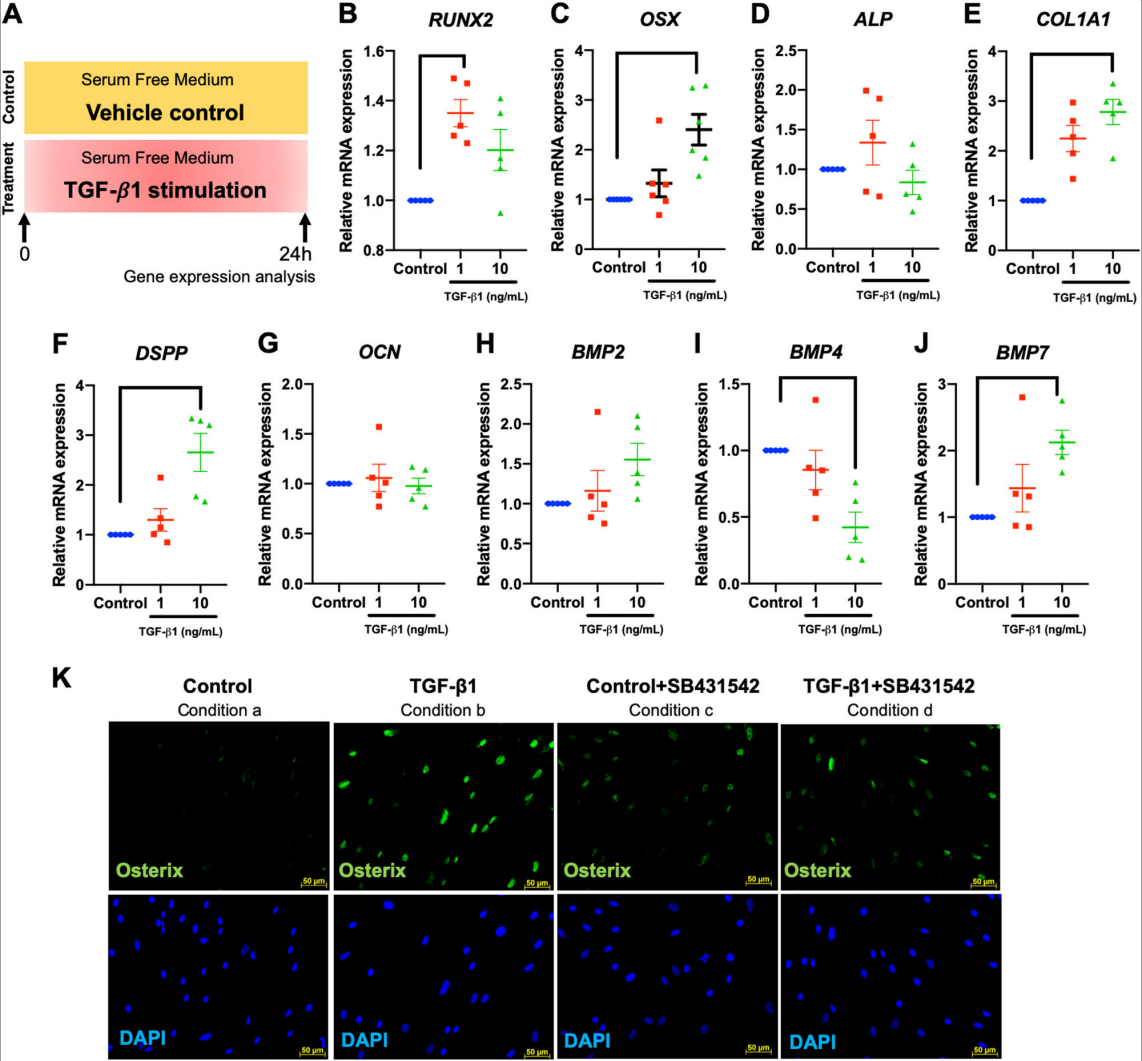
Recombinant human TGF-β1 promoted osteogenic marker gene expression. Cells were treated with 1 and 10 ng/mL of recombinant human TGF-β1 in serum-free culture medium for 24 h. In the control condition, cells were cultured in serum-free condition and the vehicle control was added in the medium. Schematic diagram of the experimental plan was illustrated (**a**). Osteogenic marker gene expression was determined using real-time polymerase chain reaction (**b**–**j**). OSX protein expression was evaluated using immunofluorescence staining (**k**). In some conditions, cells were pretreated with SB431542 30 min prior to TGF-β1 exposure. Experimental conditions were illustrated in Fig. 2i. Bars indicate a significant difference between conditions. Scale bars indicate 50 μm

To determine the influence of TGF-β1 on hPDL osteogenic differentiation, the expression of osteogenic marker genes was evaluated after cells were exposed to various concentrations of recombinant human TGF-β1 in serum-free medium for 24 h. In the control condition, cells were cultured in serum-free condition and the vehicle control was added in the medium. Schematic diagram of the experimental plan was illustrated (Fig. 7a). TGF-β1 increased the expression of *RUNX2*, *OSX*, *COL1A1*, *DSPP*, and *BMP7* mRNA levels, while attenuating *BMP4* mRNA expression (Fig. 7b–j). The increased expression of OSX protein was markedly observed after cells were exposed to TGF-β1 (10 ng/mL) and this effect was attenuated by pretreatment with a TGF-β receptor inhibitor (SB431542) (Fig. 7k), suggesting the involvement of TGF-β signaling in OSX expression in hPDLs. Experimental conditions of inhibitor experiment were illustrated in Fig. 2i.

**Fig. 8:**
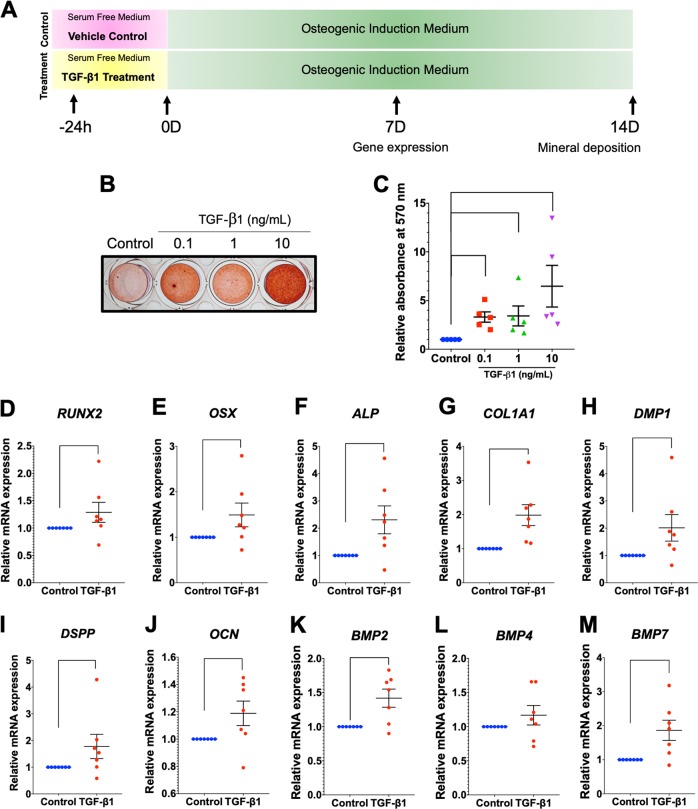
Priming with recombinant human TGF-β1 promoted osteogenic differentiation in hPDLs. Schematic diagram of the experimental plan of TGF-β1 priming and subsequent osteogenic induction (**a**). Mineralization was examined using alizarin red s staining at day 14 after osteogenic induction (**b**). The normalized absorbance of alizarin red dye (**c**). Osteogenic marker gene expression was determined using real-time polymerase chain reaction at day 7 after osteogenic induction (**d**–**m**). Bars indicate a statistically significant difference between conditions

Rather than using ICF pretreatment, hPDLs were exposed to recombinant human TGF-β1 for 24 h in serum-free medium and subsequently maintained in osteogenic medium for 14 days (Fig. 8a). In control condition, cells were treated with vehicle control in serum-free condition and further maintained in osteogenic medium (Fig. 8a). TGF-β1 pretreatment dramatically induced mineral deposition in a dose-dependent manner (Fig. 8b, c). A significant difference in mineralization was observed at all concentrations of TGF-β1 pretreatment compared with the control (Fig. 8c). In addition, pretreatment with 10 ng/mL TGF-β1 significantly upregulated *RUNX2*, *OSX*, *ALP*, *COL1A1*, *DMP1*, *DSPP*, *OCN*, *BMP2*, and *BMP7* mRNA levels after 7 days in osteogenic medium (Fig. 8d–m).

**Fig. 9:**
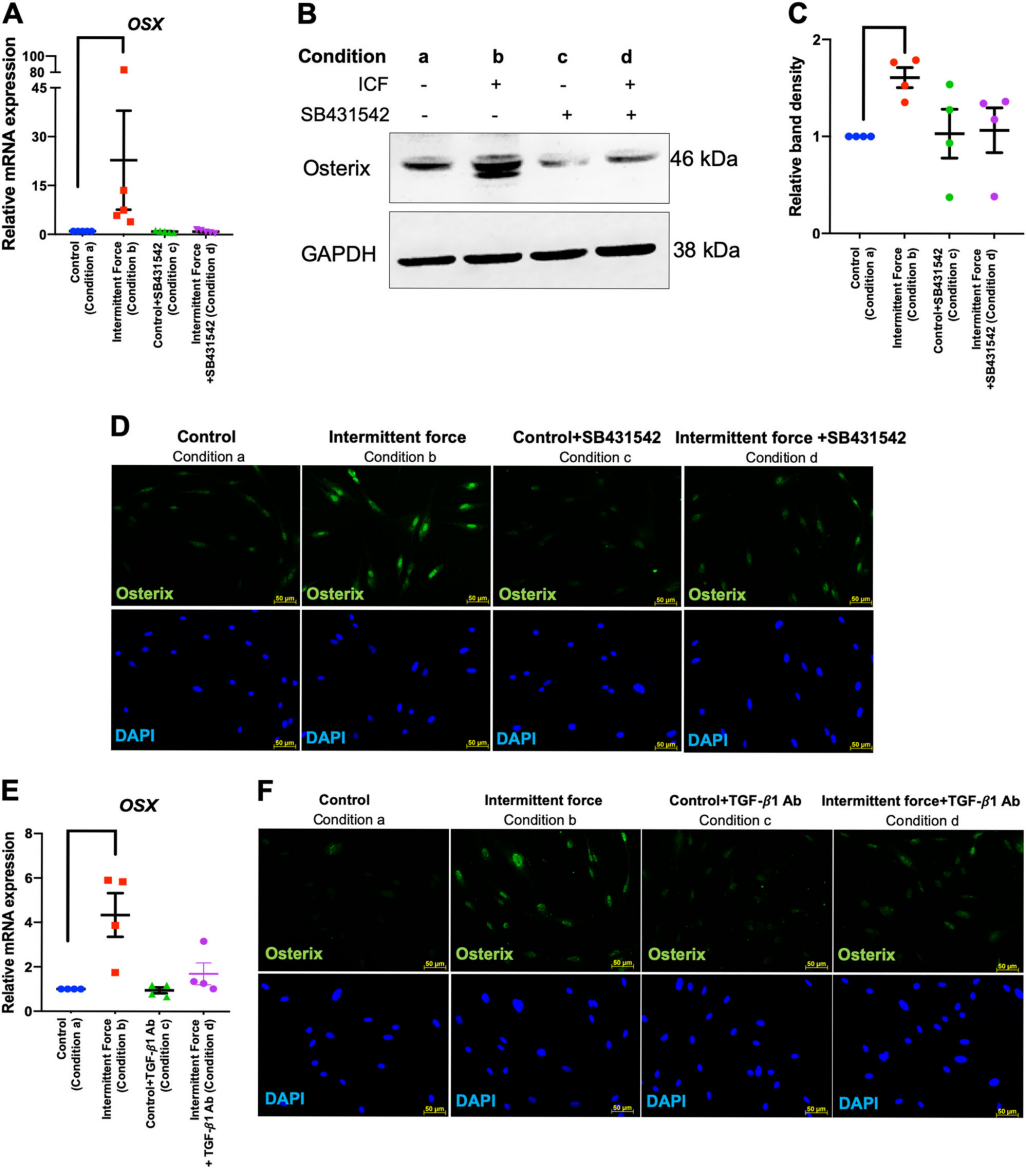

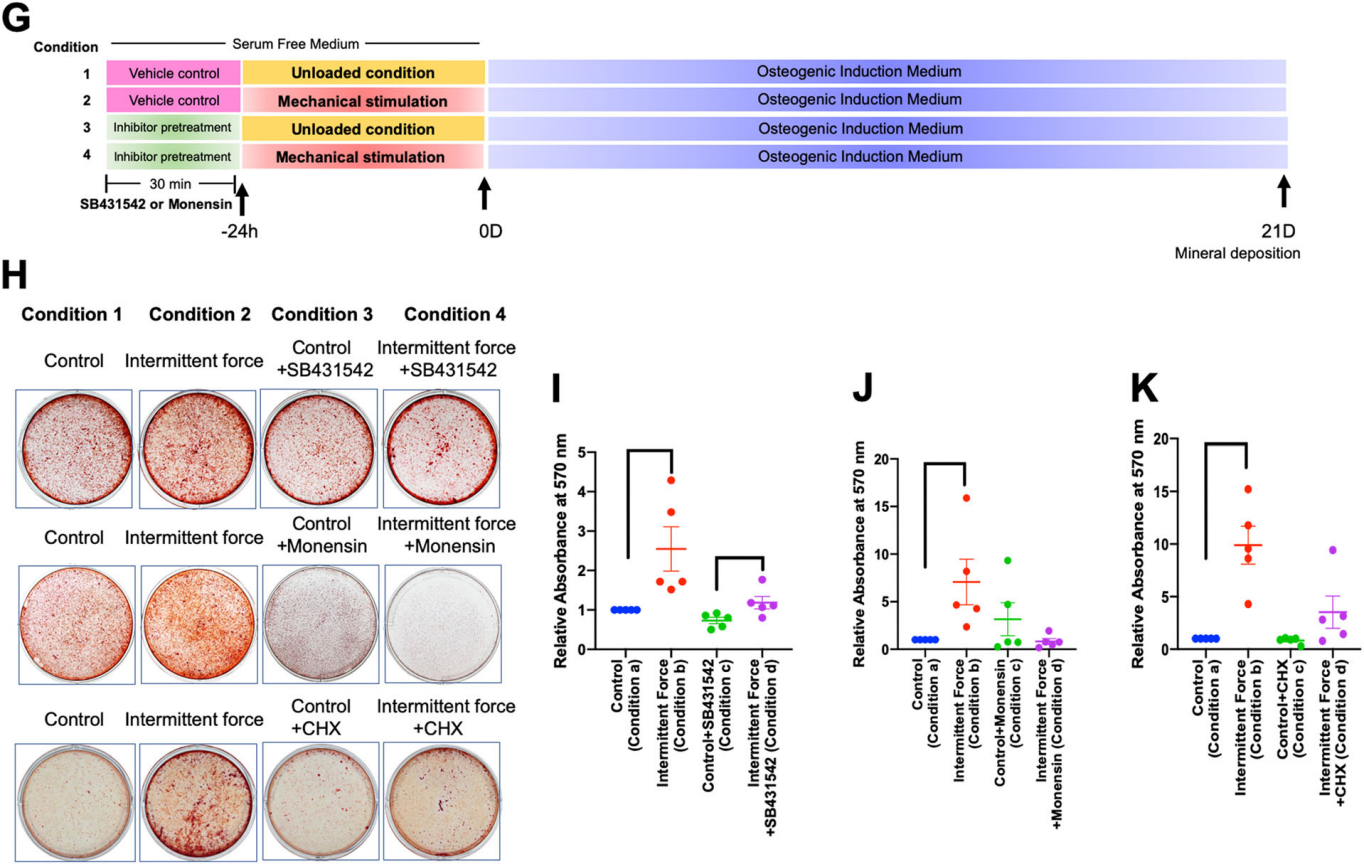
TGF-β1 participated in the ICF-induced osteogenic differentiation in hPDLs. Cells were pretreated with SB431542 or TGF-β1 neutralizing antibody for 30 min prior to ICF stimulation for 24 h in serum-free culture medium. Experimental conditions were illustrated in Fig. 2i. *OSX* mRNA expression was evaluated using real-time polymerase chain reaction (**a**, **e**). OSX protein expression was demonstrated using western blot (**b**) and immunofluorescence staining (**d**, **f**). The normalized band density was illustrated (**c**). Schematic diagram of the experimental plan for evaluating the influence of TGF-β1 on mineralization in hPDLs was demonstrated (**g**). Cells were exposed with SB431542 or monensin or cycloheximide for 30 min prior to ICF stimulation in serum-free culture medium for 24 h and subsequently maintained in osteogenic medium. Mineral deposition was examined using alizarin red s staining at day 21 after osteogenic induction (**h**). The relative absorbance of the solubilized alizarin red dye was demonstrated (**i–k**). Bars indicate a significant difference between conditions. Scale bars indicated 50 μm

The role of TGF-β signaling in the ICF-induced osteogenic differentiation in hPDLs was determined. Pretreatment with SB431542 or neutralizing antibodies against TGF-β1 attenuated the ICF-induced *OSX* mRNA and protein expression at 24 h in serum-free medium (Fig. 9a–f). Cells were pretreated with SB431542 30 min prior to ICF treatment in serum-free medium for 24 h and subsequently the cells were maintained in osteogenic medium for 21 days (Fig. 9g). SB431542 pretreatment prior to force stimulation attenuated the mineral deposition compared with the ICF-treated group (Fig. 9h, i).

As mentioned above, we demonstrated that monensin inhibited the effect of ICF on *TGFB1* mRNA expression. We then further demonstrated that monensin pretreatment prior to ICF stimulation also inhibited mineral deposition (Fig. 9h, j). Moreover, cycloheximide was used to investigate whether the TGF-β1-induced OSX expression participates in the regulation of hPDL differentiation. Cells were pretreated with cycloheximide for 30 min prior to ICF stimulation for 24 h and subsequently maintained in osteogenic medium. Cycloheximide pretreatment attenuated the ICF pretreatment-stimulated mineral deposition (Fig. 9h, k).

## Discussion

The present study demonstrated that pretreatment with ICF, but not CCF, promoted hPDL osteogenic differentiation and mineral deposition. We also found that the TGF-β signaling pathway participated in the ICF-induced osteogenic differentiation, and that ICF significantly increased *TGFB1* expression. Moreover, inhibiting the TGF-β pathway attenuated the ICF-induced OSX expression. Lastly, pretreatment with a TGF-β receptor inhibitor prior to ICF abolished the ICF pretreatment-induced mineralization. These data implicate the TGF-β signaling pathway in the promotion of osteogenic differentiation by mechanical force pretreatment.

Mechanical stimulation, such as shear, tensile, centrifugation, and vibration, has been shown to regulate osteogenic differentiation^28–32^. Cyclic tensile strain significantly enhanced RUNX2 and OSX expression in human periodontal ligament stem cells (hPDLSCs)^33^. Cyclic stretching upregulated ALP enzymatic activity and osteogenic marker gene expression through the YAP, ROCK, and myosin remodeling pathways^32^. In addition, Wnt signaling also contributed in the hydrostatic force-induced ALP enzymatic activity as well as *RUNX2* and *OSX* mRNA expression in hPDLSCs^31^. Thus, these data indicate that different types of force application may utilize different pathways to control osteogenic differentiation in hPDLs. The effect of CCF and ICF on osteogenic differentiation potency of hPDLs remains unknown. The present study illustrated that ICF treatment in serum-free culture medium for 24 h led to significantly increased OSX expression. Pretreatment with ICF prior to osteogenic induction promoted *OSX*, *COL1A1*, *DSPP*, *BMP2*, and *BMP7* mRNA expression as well as mineral deposition by hPDLs. Conversely, CCF treatment inhibited OSX expression and CCF pretreatment did not markedly influence osteogenic differentiation. This information confirms that different force applications differentially regulate hPDL differentiation.

Mechanical force is known to stimulate ATP release from hPDLs. It has been shown that centrifugation-mediated force application resulted in ATP release from hPDLs^34^. Our results also showed that both ICF and CCF dramatically stimulated ATP release from hPDLs. Mechanistically, mechanical force stimulated ATP release via connexin43 and the intracellular calcium signaling pathway^26^. It has been reported that ATP supplementation in osteogenic medium enhanced osteogenic marker gene expression and mineralization in several cell types^27,35–37^. In the present study, short-term ATP treatment prior to osteogenic induction did not influence osteogenic marker gene expression or mineralization by hPDLs. Hence, short-term treatment with ATP may not promote osteogenic differentiation in this cell type. However, continuous long-term ATP supplementation in osteogenic medium may affect the osteogenic ability of hPDLs. Therefore, further investigation is required on this aspect.

Mechanical stimulation regulates a global change in the gene-expression profile in hPDLs. Although several studies investigated mRNA profiles, the comparison of gene expression in hPDLs subjected to different force types is lacking^10,38,39^. Uniaxial cyclic strain application induced genes related to cell cycle, apoptosis, and proliferation in hPDLs^40^. In contrast, static compressive force regulated genes related to the ECM, inflammatory cytokines, and cell growth^10^. These results indicate that different force types differentially regulate global gene expression patterns in hPDLs. However, a comparison among/between different force types has not yet been reported. In addition, the influence of ICF on the global gene expression in hPDLs remains unknown. In the present study, the mRNA expression profile was compared between CCF and ICF stimulation in hPDLs. The overall results suggest that ICF exhibits more influence on hPDL gene expression compared with CCF treatment. For the same genes, the effect of ICF on mRNA expression levels is more robust compared with CCF treatment. This direct comparison confirms the distinct biological effects of specific force types on hPDL behavior.

ICF differentially regulated more genes in TGF-β signaling pathway in hPDLs compared with CCF treatment. Rat periodontal tissues exhibited high Tgf-β1 expression compared with dental pulp tissues and alveolar bone^41^. Furthermore, rodent periodontal ligament cells expressed higher mRNA levels of *Tgfb1* and *Tgfb3* than that of *Tgfb2*^42,43^. A study in rat periodontal ligament cells revealed that during osteogenic differentiation in vitro, *Tgfb1* mRNA levels increased in a time-dependent manner^43^. Conversely, *Tgfb3* was downregulated^43^. These observations imply a role for TGF-β1 in osteogenic differentiation in PDLs. It has been demonstrated that the TGF-β signaling pathway negatively regulated early osteogenic commitment in a murine periodontal ligament cell line (MPDL22), as treatment with a TGF-β receptor kinase inhibitor enhanced BMP-2-induced mineralization^42^. However, inhibiting TGF-β signaling alone in osteogenic medium without BMP-2 stimulation did not affect mineral deposition by this murine cell line^42^. Treating hPDLs twice with TGF-β1 attenuated ALP activity and osteogenic marker gene expression via the reduction of IGF-1 and pAkt^44,45^. In contrast, a single TGF-β1 treatment enhanced *RUNX2*, *ALP*, and *IGF1* mRNA expression and ALP enzymatic activity^45,46^. Corresponding with the present study, TGF-β1 pretreatment for 24 h in serum-free medium before osteogenic induction promoted osteogenic differentiation in hPDLs as shown by a significant increase in mineralization and osteogenic marker gene expression. Considering all of this evidence together, TGF-β signaling has complex regulatory effects. This signaling negatively and positively controls osteogenic differentiation in hPDLs depending on the duration and differentiation stage during TGF-β1 exposure.

The present study demonstrated that 45 and 12 genes in the focal adhesion pathway were differentially expressed by ICF and CCF compressive treatment, respectively. A previous report demonstrated that tensile force stimulated the formation of actin stress fibers in hPDLs^47^. Mechanical force regulated the expression of molecules related to bone resorption (OPG, RANKL, and M-CSF) and inflammatory-related mediator (TNF-α, PGE2, and COX2) expression in hPDLs through the integrin-focal adhesion kinase (FAK) pathway^48,49^. Moreover, inhibiting FAK attenuated the tensile force-induced ALP enzymatic activity and OCN expression^47^. Although the present study illustrated the role of TGF-β signaling pathway in ICF-pretreatment-stimulated osteogenic differentiation in hPDLs, the influence of the focal adhesion pathway cannot be excluded. The involvement/interaction of TGF-β and focal adhesion pathways in ICF-induced osteogenic gene expression and mineralization should be further determined.

Mechanical stretching regulated genes in the ECM and adhesion molecule pathways^9^. Our study demonstrated that ICF induced a more than 100-fold increase in *ITGB6* mRNA expression compared with the unloaded condition, implying the potential participation of ICF in controlling hPDL behavior. In a mouse dental papilla mesenchymal cell line, integrin β6 bound to dentin sialoprotein and subsequently induced cell attachment, migration, and *Dmp1* and *Dspp* mRNA expression^50^, suggesting that integrin β6 is involved in the regulation of dental mesenchymal cell behavior. TGF-β1 positively regulated *ITGB6* expression in oral cancer cells and gingival keratinocytes that may participate in cancer invasion and periodontal inflammation, respectively^51,52^. Our RNA sequencing data revealed increased *ITGB6* mRNA levels concurrent with the upregulation of *TGFB1* mRNA in ICF treatment. Hence, the interaction of these two molecules and their contributions to ICF pretreatment-induced osteogenic differentiation in hPDLs should be further evaluated.

In the present study, ICF significantly upregulated *OSX* and *DMP1*. We hypothesize that *OSX* might be a key regulatory factor in ICF-pretreatment-induced osteogenic differentiation. Osterix is an essential transcription factor regulating bone formation and can be controlled via a Runx2-independent pathway^53,54^. Osterix modulates the expression of various osteogenic marker genes^55^. *Osx* conditional knockout mice exhibited decreased Dmp1 and Dspp expression, which inhibited odontoblast differentiation^56^. The conditional deletion of *Osx* in dental mesenchyme resulted in reduced Dmp1 expression and cellular cementum formation^57^. Thus, OSX was chosen for investigation in the inhibitor experiments. However, the direct regulation of OSX on downstream osteogenic marker gene expression and mineral deposition under ICF stimulation in hPDLs requires further investigation.

**Fig. 10:**
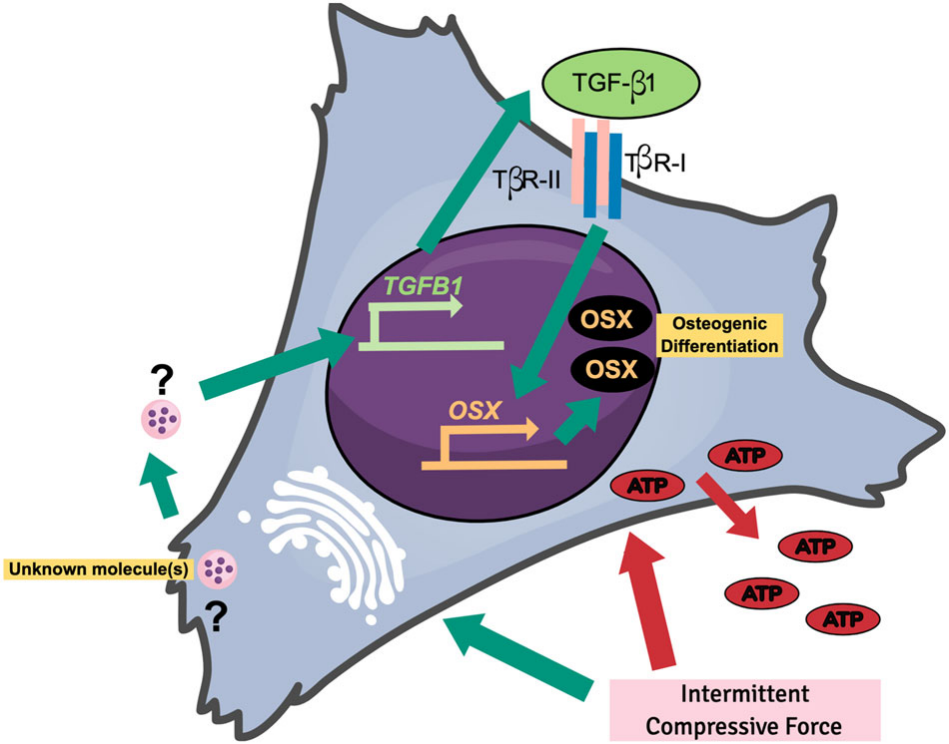
Schematic diagram of the potential regulation of OSX expression under ICF in hPDLs. ICF induces the release of molecule(s) from the golgi apparatus and subsequently enhances *TGFB1* expression. Further, TGF-β1 is released and binds to TGF-β receptors, leading to the induction of OSX expression

ICF-induced OSX expression in hPDLs and this effect was inhibited by pretreatment with cycloheximide or monensin. Cycloheximide is a protein translation inhibitor. The attenuation of ICF-induced OSX expression by cycloheximide could imply the involvement of intermediate molecule(s). ICF also promoted the expression of TGFB1 mRNA and protein. However, cycloheximide failed to inhibit the ICF-induced *TGFB1* mRNA, but not TGF-β1 protein, expression indicating an effect of ICF on TGF-β1 expression. The expression of *OSX* and *TGFB1* under ICF was abolished by pretreating the cells with monensin, an inhibitor of intracellular protein transportation and secretion from the Golgi complex, implying similar regulatory pathway(s) of *OSX* and *TGFB1* expression under ICF. Inhibiting TGF-β signaling using a chemical inhibitor (SB431542) or a TGF-β1 neutralizing antibody impeded ICF-induced OSX expression. Adding exogenous TGF-β1 also stimulated OSX expression. These results imply that TGF-β1 would be the upstream regulator of *OSX*. Further, exogenous TGF-β1 pretreatment enhanced osteogenic marker gene expression and mineralization. Correspondingly, SB431542 pretreatment prior to ICF stimulation attenuated the force-induced mineralization. Taking all this evidence together (Fig. 10), we summarize that ICF promoted the secretion of molecule(s) from Golgi complex and subsequently stimulates TGF-β1 mRNA and protein expression. Further, TGF-β1 could act in autocrine or paracrine manner to promote OSX expression, leading to the enhancement of osteogenic differentiation in hPDLs. ICF also promote ATP release but this mechanism does not involve in ICF-induced osteogenic differentiation in hPDLs.

## Supplementary information

Supplementary Tables and Figures
