# Supplementary material for: Intermittent compressive force promotes osteogenic differentiation in human periodontal ligament cells by regulating the transforming growth factor-β pathway
Source: Cell Death Dis. 2019 Oct 7;10(10):761. doi: 10.1038/s41419-019-1992-4 (PMC6779887; doi:10.1038/s41419-019-1992-4)
Supplement: Supplementary file 1 — Supplementary Tables and Figures [file 41419_2019_1992_MOESM1_ESM.docx]

**Supplementary Information**

**Intermittent compressive force promotes osteogenic differentiation in human periodontal ligament cells by regulating transforming growth factor-β** **pathway**

Jeeranan Manokawinchoke^1^, Prasit Pavasant^1^, Chenphop Sawangmake^2^, Nuttapol Limjeerajarus^3^, Chalida N. Limjeerajarus^1,4^, Hiroshi Egusa^5,*^, Thanaphum Osathanon^1,6,*^

^1^Center of Excellence for Regenerative Dentistry and Department of Anatomy, Faculty of Dentistry, Chulalongkorn University, Bangkok, 10330 Thailand

^2^Department of Pharmacology, Faculty of Veterinary Science, Chulalongkorn University, Bangkok, 10330 Thailand

^3^Research Center for Advanced Energy Technology, Faculty of Engineering, Thai-Nichi Institute of Technology, Bangkok 10250 Thailand

^4^Department of Physiology, Faculty of Dentistry, Chulalongkorn University, Bangkok, 10330 Thailand

^5^Division of Molecular and Regenerative Prosthodontics, Tohoku University Graduate School of Dentistry, Sendai 980-8575, JAPAN

^6^Genomics and Precision Dentistry Research Unit, Faculty of Dentistry, Chulalongkorn University, Bangkok, 10330 Thailand

**Co-corresponding author**

Thanaphum Osathanon, DDS, PhD

Department of Anatomy, Faculty of Dentistry,

Chulalongkorn University, Bangkok, 10330 Thailand

Tel: +66-2-218-8885

Fax: +66-2-218-8870

Email: [thanaphum.o@chula.ac.th](mailto:thanaphum.o@chula.ac.th)

Hiroshi Egusa, DDS, PhD

Division of Molecular and Regenerative Prosthodontics,

Tohoku University Graduate School of Dentistry,

Sendai 980-8575, JAPAN

Email: [egu@dent.tohoku.ac.jp](mailto:egu@dent.tohoku.ac.jp)

**Supplementary Table 1** Oligonucleotide sequences

| **Gene** | **Primer sequences (F=forward, R=Reverse)** | | **Accession number** |
| --- | --- | --- | --- |
| ***GAPDH*** | F | 5’-TCA TGG GTG TGA ACC ATG AGA A-3’ | NM_002046.3 |
|  | R | 5’-GGC ATG GAC TGT GGT CAT GAG-3’ |  |
| ***RUNX2*** | F | 5’-ATG ATG ACA CTG CCA CCT CTG A-3’ | NM_001024630.3 |
|  | R | 5’-GGC TGG ATA GTG CAT TCG TG-3’ |  |
| ***OSX*** | F | 5’-GCC AGA AGC TGT GAA ACC TC-3’ | NM_152860.1 |
|  | R | 5’-GCT GCA AGC TCT CCA TAA CC-3’ |  |
| ***ALP*** | F | 5’-CGA GAT ACA AGC ACT CCC ACT TC-3’ | NM_000478.3 |
|  | R | 5’-CTG TTC AGC TCG TAC TGC ATG TC-3’ |  |
| ***COL1A1*** | F | 5’-GTG CTA AAG GTG CCA ATG GT-3’ | NM_000088.3 |
|  | R | 5’-ACC AGG TTC ACC GCT GTT AC-3’ |  |
| ***DMP1*** | F | 5’-CAG GAG CAC AGG AAA AGG AG-3’ | NM_004407.3 |
|  | R | 5’-CTG GTG GTA TCT TGG GCA CT-3’ |  |
| ***DSPP*** | F | 5’-ATA TTG AGG GCT GGA ATG GGG A-3’ | NM_014208.3 |
|  | R | 5’-TTT GTG GCT CCA GCA TTG TCA-3’ |  |
| ***OCN*** | F | 5’-CTT TGT GTC CAA GCA GGA GG-3’ | NM_199173.4 |
|  | R | 5’-CTG AAA GCC GAT GTG GTC AG-3’ |  |
| ***BMP2*** | F | 5’-GCG TGA AAA GAG AGA CTG C-3’ | NM_001200.2 |
|  | R | 5’-CCA TTG AAA GAG CGT CCA C-3’ |  |
| ***BMP4*** | F | 5’-AAA GTC GCC GAG ATT CAG GG-3’ | NM_001202.4 |
|  | R | 5’-GAC GGC ACT CTT GCT AGG C-3’ |  |
| ***BMP7*** | F | 5’-TCG GCA CCC ATG TTC ATG C-3’ | NM_001719.2 |
|  | R | 5’-GAG GAA ATG GCT ATC TTG CAG G |  |
| ***TGFB1*** | F | 5’-GGA TAC CAA CTA TTG CTT CAG CTC C-3’ | NM_000660.4 |
|  | R | 5’-AGG CTC CAA ATG TAG GGG CAG GGC C-3’ |  |
| ***TGFB2*** | F | 5’-ATT GCC CTC CTA CAG ACT TGA G-3’ | NM_001135599.2 |
|  | R | 5’-CAG CAC AGA AGT TGG CAT TGT A-3’ |  |
| ***TGFB3*** | F | 5’-GGT TTT CCG CTT CAA TGT GT-3’ | NM_003239.2 |
|  | R | 5’-GCT CGA TCC TCT GCT CAT TC-3’ |  |

**Supplementary Table 2** Top 20 significantly upregulated and downregulated genes in the CCF-treated hPDLs.

| Gene symbol | Gene name | Log2FC | | FDR |  |
| --- | --- | --- | --- | --- | --- |
| Upregulated genes | | | | | |
| *AMIGO2* | Adhesion molecule with Ig like domain 2 | 3.65 | | 2.57E-74 |  |
| *FZD8* | Frizzled class receptor 8 | 4.33 | | 2.79E-69 |  |
| *KRT16* | Keratin 16 | 5.43 | | 3.84E-45 |  |
| *CSRP2* | Cysteine and glycine rich protein 2 | 2.34 | | 3.87E-37 |  |
| *DACT1* | Disheveled binding antagonist of beta catenin 1 | 2.41 | | 5.95E-34 |  |
| *NREP* | Neuronal regeneration related protein | 2.83 | | 3.08E-32 |  |
| *LMCD1* | LIM and cysteine rich domains 1 | 3.16 | | 1.00E-29 |  |
| *KCNG1* | Potassium voltage-gated channel modifier subfamily G member 1 | 2.43 | | 4.48E-25 |  |
| *FSTL3* | Follistatin-like 3 (secreted glycoprotein) | 2.00 | | 8.67E-25 |  |
| *TSPAN2* | Tetraspanin 2 | 4.15 | | 8.92E-22 |  |
| *CCDC99* | Coiled-coil domain containing 99 | 2.50 | | 1.18E-21 |  |
| *EGR2* | Early growth response 2 | 4.07 | | 1.18E-21 |  |
| *LTBP1* | Latent transforming growth factor beta binding protein 1 | 2.11 | | 1.19E-21 |  |
| *PMEPA1* | Prostate transmembrane protein, androgen induced 1 | 2.26 | | 2.01E-21 |  |
| *SKIL* | SKI-like oncogene | 1.99 | | 6.31E-21 |  |
| *TPM1* | Tropomyosin 1 (alpha) | 2.44 | | 2.52E-20 |  |
| *LRRC15* | Leucine rich repeat containing 15 | 2.78 | | 5.63E-20 |  |
| *SLC2A1* | Solute carrier family 2 (facilitated glucose transporter), member 1 | 2.09 | | 4.01E-19 |  |
| *LIMS1* | LIM and senescent cell antigen-like domains 1 | 1.66 | | 1.36E-18 |  |
| *BHLHE40* | Basic helix-loop-helix family, member e40 | 2.31 | | 1.67E-18 |  |
| Downregulated genes | | | | | |
| *SECTM1* | Secreted and transmembrane 1 | -1.69 | 8.02E-15 | |  |
| *RYR2* | Ryanodine receptor 2 | -2.59 | 9.29E-15 | |  |
| *ADH1B* | Alcohol dehydrogenase 1B (class I), beta polypeptide | -4.90 | 2.39E-13 | |  |
| *CNKSR3* | CNKSR family member 3 | -1.48 | 2.49E-13 | |  |
| *NEDD4L* | Neural precursor cell expressed, developmentally down-regulated 4-like, E3 ubiquitin protein ligase | -1.35 | 2.99E-13 | |  |
| *SLC9A9* | Solute carrier family 9 (sodium/hydrogen exchanger), member 9 | -1.38 | 8.50E-13 | |  |
| *RSPO2* | R-spondin 2 | -2.59 | 1.16E-12 | |  |
| *EPHA4* | EPH receptor A4 | -1.58 | 7.90E-11 | |  |
| *PPAP2B* | Phosphatidic acid phosphatase type 2B | -1.75 | 1.11E-10 | |  |
| *ENPP2* | Ectonucleotide pyrophosphatase/phosphodiesterase 2 | -2.24 | 1.29E-10 | |  |
| *EYA1* | Eyes absent homolog 1 (Drosophila) | -1.12 | 1.70E-09 | |  |
| *MASP1* | Mannan-binding lectin serine peptidase 1 (C4/C2 activating component of Ra-reactive factor) | -1.34 | 2.99E-09 | |  |
| *RAB27B* | RAB27B, member RAS oncogene family | -1.11 | 1.59E-08 | |  |
| *C10orf54* | Chromosome 10 open reading frame 54 | -1.21 | 7.19E-08 | |  |
| *PDGFD* | Platelet derived growth factor D | -1.29 | 1.12E-07 | |  |
| *PTGFR* | Prostaglandin F receptor (FP) | -1.15 | 1.17E-07 | |  |
| *CLDN11* | Claudin 11 | -2.65 | 2.28E-07 | |  |
| *PTPLAD2* | Protein tyrosine phosphatase-like A domain containing 2 | -1.11 | 3.18E-07 | |  |
| *OSR1* | Odd-skipped related 1 (Drosophila) | -1.54 | 3.19E-07 | |  |
| *FAM43A* | Family with sequence similarity 43, member A | -1.58 | 4.55E-07 | |  |

**Supplementary Table 3** Top 20 significantly upregulated and downregulated genes in the ICF-treated hPDLs.

| Gene symbol | Gene name | Log2FC | | FDR |  |
| --- | --- | --- | --- | --- | --- |
| Upregulated genes | | | | | |
| *AMIGO2* | Adhesion molecule with Ig-like domain 2 | 5.03 | | 6.30E-85 |  |
| *TSPAN2* | Tetraspanin 2 | 6.98 | | 2.00E-61 |  |
| *KCNG1* | Potassium voltage-gated channel, subfamily G, member 1 | 3.90 | | 3.09E-59 |  |
| *C18orf1* | Chromosome 18 open reading frame 1 | 7.67 | | 3.69E-58 |  |
| *FZD8* | Frizzled family receptor 8 | 5.46 | | 6.26E-58 |  |
| *MURC* | Muscle-related coiled-coil protein | 5.06 | | 2.14E-56 |  |
| *KCNN4* | Potassium intermediate/small conductance calcium-activated channel, subfamily N, member 4 | 3.67 | | 5.57E-50 |  |
| *KRT16* | Keratin 16 | 6.24 | | 8.37E-49 |  |
| *FABP3* | Fatty acid binding protein 3, muscle and heart (mammary-derived growth inhibitor) | 4.64 | | 9.10E-49 |  |
| *KANK4* | KN motif and ankyrin repeat domains 4 | 8.63 | | 7.81E-45 |  |
| *FSTL3* | Follistatin-like 3 (secreted glycoprotein) | 3.82 | | 4.40E-42 |  |
| *PMEPA1* | Prostate transmembrane protein, androgen induced 1 | 3.82 | | 1.14E-39 |  |
| *ZNF365* | Zinc finger protein 365 | 4.95 | | 1.40E-39 |  |
| *TIMP3* | TIMP metallopeptidase inhibitor 3 | 3.56 | | 1.71E-38 |  |
| *TRIB1* | Tribbles homolog 1 (Drosophila) | 3.90 | | 4.55E-37 |  |
| *COMP* | Cartilage oligomeric matrix protein | 6.42 | | 6.32E-37 |  |
| *NFATC2* | Nuclear factor of activated T-cells, cytoplasmic, calcineurin-dependent 2 | 4.02 | | 7.12E-37 |  |
| *PRG4* | Proteoglycan 4 | 8.25 | | 5.80E-36 |  |
| *EGR2* | Early growth response 2 | 5.83 | | 5.48E-33 |  |
| *LDLR* | Low density lipoprotein receptor | 3.42 | | 1.59E-32 |  |
| Downregulated genes | | | | | |
| *MASP1* | Mannan-binding lectin serine peptidase 1 (C4/C2 activating component of Ra-reactive factor) | -3.36 | 2.26E-43 | |  |
| *RAB27B* | RAB27B, member RAS oncogene family | -3.53 | 7.04E-40 | |  |
| *SEPP1* | Selenoprotein P, plasma, 1 | -4.40 | 1.74E-38 | |  |
| *PTX3* | Pentraxin 3, long | -3.31 | 9.99E-37 | |  |
| *PTPLAD2* | Protein tyrosine phosphatase-like A domain containing 2 | -3.08 | 2.80E-36 | |  |
| *RYR2* | Ryanodine receptor 2 (cardiac) | -5.20 | 1.73E-34 | |  |
| *RSPO2* | R-spondin 2 | -5.23 | 1.63E-33 | |  |
| *SLC2A12* | Solute carrier family 2 (facilitated glucose transporter), member 12 | -4.45 | 1.41E-32 | |  |
| *NEDD4L* | Neural precursor cell expressed, developmentally down-regulated 4-like, E3 ubiquitin protein ligase | -2.73 | 2.68E-32 | |  |
| *VWA5A* | von Willebrand factor A domain containing 5A | -3.90 | 2.97E-32 | |  |
| *SECTM1* | Secreted and transmembrane 1 | -4.97 | 1.15E-31 | |  |
| *PPAP2B* | Phosphatidic acid phosphatase type 2B | -3.14 | 1.65E-31 | |  |
| *RCAN2* | Regulator of calcineurin 2 | -3.08 | 3.20E-31 | |  |
| *CASP1* | Caspase 1, apoptosis-related cysteine peptidase | -4.98 | 9.03E-31 | |  |
| *CNKSR3* | CNKSR family member 3 | -2.82 | 9.38E-28 | |  |
| *PDGFD* | Platelet derived growth factor D | -2.78 | 4.42E-24 | |  |
| *PDGFRA* | Platelet-derived growth factor receptor, alpha polypeptide | -2.40 | 2.13E-23 | |  |
| *DAPK1* | Death-associated protein kinase 1 | -4.45 | 4.44E-22 | |  |
| *THRB* | Thyroid hormone receptor, beta | -3.09 | 1.97E-21 | |  |
| *COLEC12* | Collectin sub-family member 12 | -3.16 | 2.59E-21 | |  |

**Supplementary Table 4** Top 10 enriched KEGG pathway of the upregulated and downregulated genes in the CCF-treated hPDLs.

| Pathway | Entrez Gene ID | FDR |  |  |
| --- | --- | --- | --- | --- |
| *Upregulated pathway* | | | |  |
| Terpenoid backbone biosynthesis | *HMGCS1; IDI1; MVD; HMGCR* | 0.0040 |  |  |
| Arginine and proline metabolism | *GLS; ALDH1B1; ODC1; P4HA3; ADC* | 0.0162 |  |  |
| ECM-receptor interaction | *ITGA11; COL4A1; THBS2; ITGB3; COMP; ITGB6* | 0.0162 |  |  |
| Arrthythmogenic right ventricular cardiomyopathy (ARVC) | *DSP; CACNG4; ITGB3; ITGA11; CTNNA2; ITGB6* | 0.0162 |  |  |
| TGF-β signaling pathway | *CDKN2B; INHBE; THBS2; LTBP1; COMP; INHBA* | 0.0162 |  |  |
| Hypertrophic cardiomyopathy (HCM) | *TPM1; CACNG4; ACTC1; ITGB3; ITGA11; ITGB6* | 0.0162 |  |  |
| Metabolic pathways | *AZIN2; DHCR7; TYMP; ALDH1B1; AMACR; GCH1; GLS; P4HA3; HMGCR; HMGCS1; IMPA2; MVD; ACLY; NNMT; ODC1; PLA2G3; PLCB4; LPCAT2; PRPS1; CYP4F11; MSMO1; XYLT1; UAP1; BPGM; SQLE; UCK2; DGKI; LIPG; PRDX6* | 0.0162 |  |  |
| Dilated cardiomyopathy | *ITGA11; CACNG4; ITGB3; ITGB6; ACTC1; TPM1* | 0.0195 |  |  |
| Steroid biosynthesis | *DHCR7; MSMO1; SQLE* | 0.0205 |  |  |
| African trypanosomiasis | *F2RL1; IL12A; PLCB4* | 0.1051 |  |  |
| *Downregulated pathway* | | | |  |
| Calcium signaling pathway | *PTGFR; PDE1A; ADRA1D; PLCD4; HTR2B; ATP2B2; RYR2; PDGFRA; SLC8A1; HRH1; EDNRA* | 2.28e-05 |  |  |
| Axon guidance | *PLXNC1; EPHA4; EFNA5; SEMA3E; SEMA3A; SEMA6A; NTNG1; SRGAP3* | 0.0005 |  |  |
| Cytokine-cytokine receptor | *CCR1; CXCR7; TGFB3; TNFSF13B; TNFS15; ACVR2B; TGFB2; PDGFRA; KIT; TNFRSF19* | 0.0011 |  |  |
| Dilated cardiomyopathy | *RYR2; LAMA2; ITGB8; TGFB2; SLC8A1; TGFB3* | 0.0011 |  |  |
| Hypertrophic cardiomyopathy | *RYR2; LAMA2; ITGB8; TGFB2; SLC8A1; TGFB3* | 0.0011 |  |  |
| TGF-β signaling pathway | *ID2; BMP4; ACVR2B; TGFB2; TGFB3* | 0.0075 |  |  |
| Neuroactive ligand-receptor interaction | *THRB; PTGFR; ADRA1D; LPAR6; HTR2B; GRIA4; EDNRA; HRH1* | 0.0208 |  |  |
| Arrthythmogenic right ventricular cardiomyopathy (ARVC) | *RYR2; LAMA2; ITGB8; SLC8A1* | 0.0273 |  |  |
| Tytosine metabolism | *MAOA; ADH1B; ALDH3A1* | 0.0330 | | |
| ABC Transporters | *ABCA6; ABCA9; ABCA8* | 0.0364 | | |

**Supplementary Table 5** Top 10 enriched KEGG pathway of the upregulated and downregulated genes in the ICF-treated hPDLs.

| Pathway | Entrez Gene ID | FDR |  |  |
| --- | --- | --- | --- | --- |
| *Upregulated pathway* | | | | |
| Pathways in cancer | *CDKN2B; NKX3-1; FZD8; SLC2A1; WNT2; XIAP; CYCS; FGF21; PDGFB; FZD6; CTNNA2; WNT5A;PDGF1; JUN; ITGA3; MYC; IGF1; COL4A1; PIK3CD; MET; FGF11; GLI1; TGFB1; NRAS; WNT9A; WNT10B; FZD4; COL4A4; WNT11; WNT7B; TCF7; VEGFA; FGF1; PPARD; PTGS2; LEF1; HIF1A; WNT5B; E2F1; RUNX1; LAMC2; CSF1R* | 2.26e-06 |  |  |
| Focal adhesion | *FLNC; PDGFC; VAV3; COL4A4; XIAP; ACTN1; VEGFA; PDGFB; PDGFA; JUN; VASP; ITGA3; ITGA7; COL2A1; MYLK; COL5A1; IGF1; ACTN4; COL4A1; ITGA11; TNN; PIK3CD; MET; ITGB6; COMP; LAMC2; MYLK2* | 6.87E-05 |  |  |
| Basal cell carcinoma | *WNT9A; LEF1; WNT10B; FZD4; WNT5B; FZD8; WNT2; TCF7; WNT11; WNT7B; FZD6; GLI1; WNT5A* | 6.87e-05 |  |  |
| Regulation of actin cytoskeleton | *PDGFC; VAV3; NRAS; EZR; MYH9; CHRM4; MRAS; ACTN1; FGF21; NCKAP1; PDGFB; FGF1; PDGFA; ITGA3; ARPC5; ITGA7; LIMK1; MYLK; SLC9A1;ACTN4; ITGA11; PIK3CD; ITGB6; TIAM1; MSN; LIMK2; MYLK2; FGF11* | 6.87e-05 |  |  |
| Melanogenesis | *CREB3L2; WNT9A; NRAS; WNT10B; FZD4; FZD8; WNT2; TCF7; WNT7B; WNT11; FZD6; WNT5A; CALM2; LEF1; WNT5B; ADCY2; ADCY7; TYRP1* | 6.87e-05 |  |  |
| Arrhythmogenic right ventricular cardiomyopathy (ARVC) | *SGCA; LEF1; CACNG4; ITGA3; ITGA7; DSG2; SGCD; ACTN4; TCF7; ITGA11; ACTN1; ITGB6; CDH2; DSP; CTNNA2* | 7.19E-05 |  |  |
| TGF-β signaling pathway | *TGFB1; CDKN2B; LTBP1; ID1; ACVR1; BMPR2; INHBB; ACVR1C; MYC; SMAD7; INHBE; COMP; INHBA; NODAL* | 0.0013 |  |  |
| Cytokine-cytokine receptor interaction | *TGFB1; CXCR5; LIF; PDGFC; IL11; ACVR1; BMPR2; CCL2; RELT; CLCF1; TNFRSF21; CSF2; VEGFA; CXCR3; PDGFB; IL21R; TNFSF9; PDGFA; IL12A; INHBB; INHBE; TNFRSF12A; MET; TNFSF4; TNFRSF10D; TNFSF18; INHBA; CSF1R* | 0.0019 |  |  |
| Wnt signaling pathway | *WNT9A; WNT10B; FZD4; PORCN; FZD8; WNT2; NFATC2; TCF7; WNT7B; WNT11; DAAM1; FZD6; WNT5A; JUN; PPARD; LEF1; MYC; WNT5B; PRICKLE2* | 0.0034 |  |  |
| Dilated cardiomyopathy | *TGFB1; SGCA; ACTC2; CACNG4; ITGA3; ADCY2; ITGA7; SGCD; IGF1; ITGA11; TPM1; ITGB6; ADCY7* | 0.0076 |  |  |
| *Downregulated pathway* | | | | |
| Calcium signaling pathway | *P2RX4; PTGFR; ITPKB; TNNC2; CYSLTR1; P2RX7; RYR2; ADORA2B; ADRB2; PDGFRA; EGFR; GNA14; HRH2; SLC8A1; EDNRA; ADRA1B; CAMK2D; PDE1A; ADRA1D; P2RX6;PLCD4; HTR2B; PTK2B; OXTR; ERBB3; PHKG1; HEH1* | 0.0013 |  |  |
| Tryptophan metabolism | *IDO1; CYP1A1; EHHADH; HAAO; INMT; AOX1; MAOA; CCBL2; ALDH7A1; GCDH; ALDH3A2;* | 0.0022 |  |  |
| Drug metabolism-cytochrome P450 | *ADH1A; GSTM5; GSTM2; FMO1; FMO5; FMO4; GSTM4; ALDH3A1; AOX1; MAOA; MGST2; ADH1B; FMO3; ADH1C* | 0.0040 |  |  |
| Pathways in cancer | *FGF13; FGF10; JUP; FGF5; PDGFRA; ITGA2B; WNT2B; DAPK2; GLI3; MITF; TGFB3; DAPK1; AXIN2; BCL2; WNT16; ARNT2; CASP8; KIT; PTCH2; LAMA4; BIRC3; PPARG; PTCH1; MMP1; RASSF5; LAMA2; EGFR; SMAD3; PGF; TCF7L1; TRAF1; FGFR2; AR; FGF7; HGF; STAT5A; BMP4; PLD1* | 0.0040 |  |  |
| Fatty acid metabolism | *ACSL5; ADH1A; ACADS; ACADSB; EHHADH; ALDH7A1; GCDH; ADH1B; ALDH3A2; ADH1C* | 0.0053 |  |  |
| Valine, leucine and isoleucine degradation | *ACADS; ACADSB; ALDH6A1; EHHADH; BCKDHB; MCCC1; AOX1; ALDH7A1; ABAT; ALDH3A2* | 0.0053 |  |  |
| Propanoate metabolism | *ACACB; ACSS3; ALDH6A1; EHHADH; ALDH7A1; ABAT; ACSS1; ALDH3A2* | 0.0114 |  |  |
| Axon guidance | *PLXNC1; EPHA5; NFATC1; SEMA6A; PLXNB1; SRGAP3; GNAI1; FYN; SEMA3D; EFNA5; NTN4; EPHB6; NTN1; SEMA3A; NTNG1; FES; SEMA6C; UNC5D* | 0.0140 |  |  |
| Arrhythmogenic right ventricular cardiomyopathy (ARVC) | *TCF7L1; ITGB8; JUP; ITGA4; RYR2; ACTN2; LAMA2; ITGAB4; CACNA2D4; CACNB4; ITGA2B; SLC8A1* | 0.0267 | |  |
| ABC transporters | *ABCA5; ABCG2; ABCA10; ABCA13; ABCA6; ABCA7; ABCA9; ABCA8* | 0.0640 | |  |

**
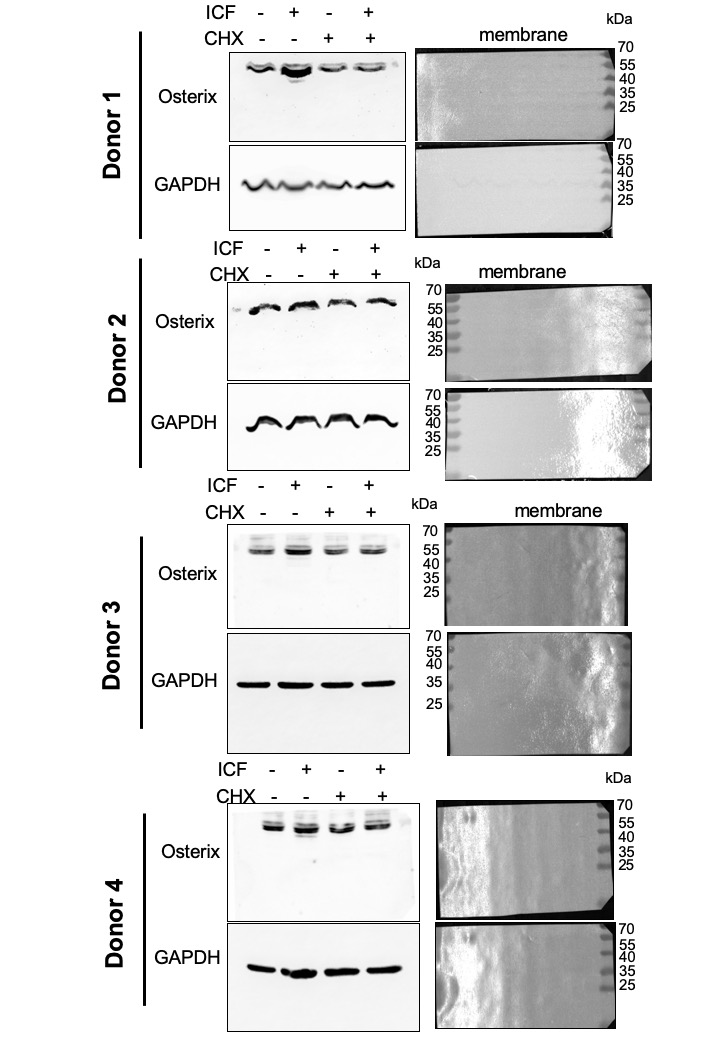
**

**Supplementary Figure 1** Cells were pretreated with cycloheximide (CHX) for 30 min prior to ICF stimulation for 24 h in serum free culture medium. OSX and GAPDH protein expression was demonstrated using western blot. Cells from four different donors were utilized.

**
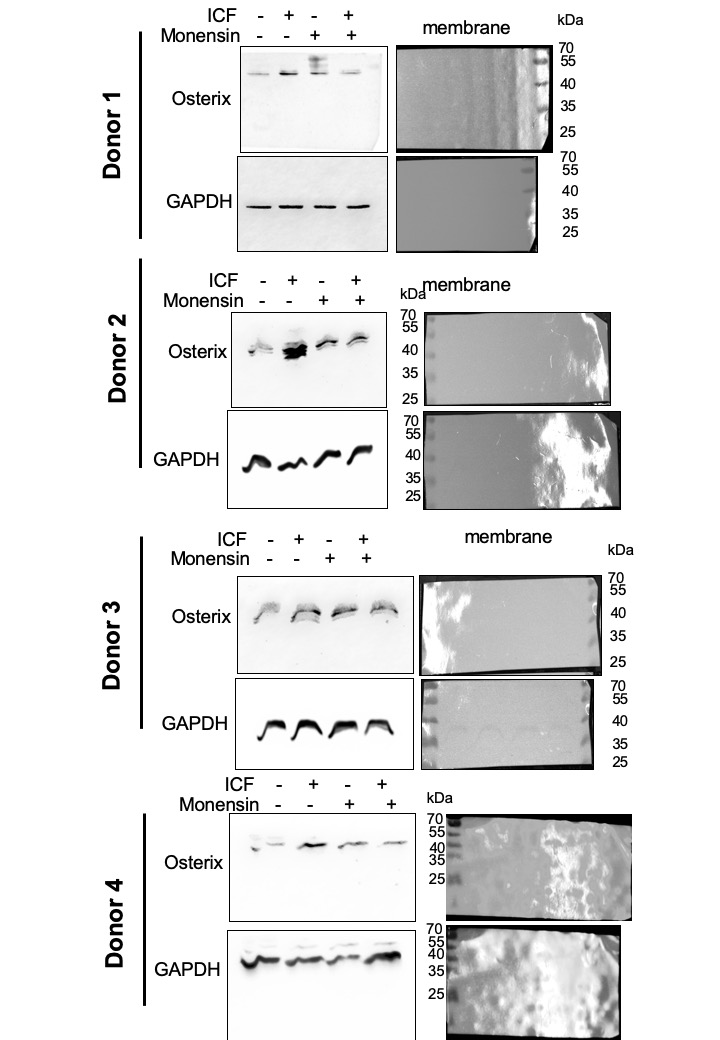
**

**Supplementary Figure 2** Cells were pretreated with monensin for 30 min prior to ICF stimulation for 24 h in serum free culture medium. OSX and GAPDH protein expression was demonstrated using western blot. Cells from four different donors were utilized.

**
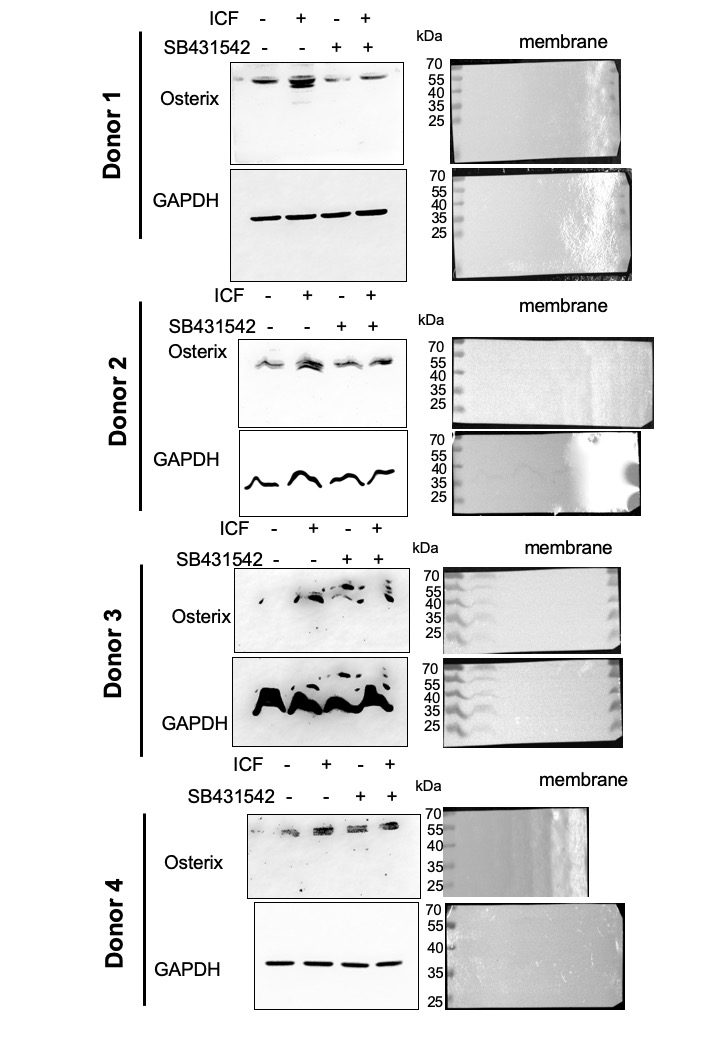
**

**Supplementary Figure 3** Cells were pretreated with SB431542 for 30 min prior to ICF stimulation for 24 h in serum free culture medium. OSX and GAPDH protein expression was demonstrated using western blot. Cells from four different donors were utilized.
